# Multiple Polymorphisms Affect Expression and Function of the Neuropeptide S Receptor (NPSR1)

**DOI:** 10.1371/journal.pone.0029523

**Published:** 2011-12-21

**Authors:** Francesca Anedda, Marco Zucchelli, Danika Schepis, Anna Hellquist, Lucia Corrado, Sandra D'Alfonso, Adnane Achour, Gerald McInerney, Alejandro Bertorello, Mikael Lördal, Ragnar Befrits, Jan Björk, Francesca Bresso, Leif Törkvist, Jonas Halfvarson, Juha Kere, Mauro D'Amato

**Affiliations:** 1 Department of Biosciences and Nutrition, Karolinska Institutet, Stockholm, Sweden; 2 Institute of Neurogenetics and Neuropharmacology - CNR, Monserrato, Italy; 3 Department of Microbiology Tumor Cell Biology, Karolinska Institutet, Stockholm, Sweden; 4 Department of Medical Sciences, University of Eastern Piedmont and IRCAD, Novara, Italy; 5 Center for Infectious Medicine, Karolinska University Hospital, Stockholm, Sweden; 6 Department of Medicine, Karolinska Institutet, Stockholm, Sweden; 7 Department for Clinical Science Intervention and Technology, Karolinska Institutet, Stockholm, Sweden; 8 Department of Internal Medicine, Örebro University Hospital, Örebro, Sweden; 9 School of Health and Medical Sciences, Örebro University, Örebro, Sweden; 10 Center for Biosciences, Karolinska Institutet, Stockholm, Sweden; 11 Department of Medical Genetics, University of Helsinki, and Folkhälsan Institute of Genetics, Helsinki, Finland; 12 Science for Life Laboratory, Stockholm, Sweden; Wayne State University, United States of America

## Abstract

**Background:**

neuropeptide S (NPS) and its receptor NPSR1 act along the hypothalamic-pituitary-adrenal axis to modulate anxiety, fear responses, nociception and inflammation. The importance of the NPS-NPSR1 signaling pathway is highlighted by the observation that, in humans, *NPSR1* polymorphism associates with asthma, inflammatory bowel disease, rheumatoid arthritis, panic disorders, and intermediate phenotypes of functional gastrointestinal disorders. Because of the genetic complexity at the *NPSR1* locus, however, true causative variations remain to be identified, together with their specific effects on receptor expression or function. To gain insight into the mechanisms leading to *NPSR1* disease-predisposing effects, we performed a thorough functional characterization of all *NPSR1* promoter and coding SNPs commonly occurring in Caucasians (minor allele frequency >0.02).

**Principal Findings:**

we identified one promoter SNP (rs2530547 [−103]) that significantly affects luciferase expression in gene reporter assays and *NPSR1* mRNA levels in human leukocytes. We also detected quantitative differences in NPS-induced genome-wide transcriptional profiles and CRE-dependent luciferase activities associated with three *NPSR1* non-synonymous SNPs (rs324981 [Ile107Asn], rs34705969 [Cys197Phe], rs727162 [Arg241Ser]), with a coding variant exhibiting a loss-of-function phenotype (197Phe). Potential mechanistic explanations were sought with molecular modelling and bioinformatics, and a pilot study of 2230 IBD cases and controls provided initial support to the hypothesis that different *cis*-combinations of these functional SNPs variably affect disease risk.

**Significance:**

these findings represent a first step to decipher *NPSR1* locus complexity and its impact on several human conditions NPS antagonists have been recently described, and our results are of potential pharmacogenetic relevance.

## Introduction

The latest member to be discovered in the family of neuropeptides is Neuropeptide S (NPS), characterized by the N-terminal amino acid sequence Ser-Phe-Arg-Asn-Gly-Val-Gly, which is identical in all animal species and represents the bioactive portion of the molecule.[1] NPS selectively binds and activates its receptor NPSR1 (neuropeptide S receptor, also known as GPR154 or G protein-coupled receptor for asthma susceptibility, GPRA), a 7-transmembrane G protein-coupled receptor (GPCR) that can induce intracellular signalling via mobilization of calcium, increase in cyclic adenosine monophospate (cAMP) levels, and the mitogen-activated protein kinase (MAPK) pathway.[2] NPS and NPSR1 are mainly expressed in specific regions of the brain such as the amygdala, the hypothalamic nucleus and the hippocampus and, from studies in rodents, they appear to modulate several biological functions including anxiety, locomotion, arousal, food intake, fear memory and drug addiction.[3]–[8] In addition, recent studies from our group have shown i) a direct effect of NPS on macrophage adherence, migration and phagocytosis of bacteria,[9] ii) the transcriptional induction of proinflammatory cytokines (interleukin 8 [IL8] and substance P [SP]) upon NPS stimulation of epithelial cells stably transfected with *NPSR1* cDNA[10] and iii) increased levels of *NPSR1* mRNA in activated leukocytes, in lipopolysaccharide (LPS)-stimulated peripheral blood mononuclear cells (PBMCs), and in inflamed tissues from patients suffering from asthma and inflammatory bowel disease (IBD).[9], [11]–[13] A role for the NPS-NPSR1 system in the modulation of neuroendocrine and immune functions along the hypothalamic-pituitary-adrenal (HPA) axis has therefore been postulated, and efforts are underway to develop NPSR1 agonists and antagonists that might be exploited for pharmaceutical use.[1]

Genetic variation at the *NPSR1* locus (MIM 608595) is associated in humans with predisposition to inflammatory diseases such as asthma (MIM 600807), inflammatory bowel disease (IBD [MIM 266600]) and rheumatoid arthritis (RA [MIM 180300]), intermediate phenotypes of functional gastrointestinal disorders, and panic disorders (MIM 167870);[11], [12], [14]–[19] that is, in several conditions where alterations of NPSR1 signalling properties or expression may have important pathogenetic consequences by way of dysregulating immune- or neuroendocrine-related NPS-NPSR1 system function(s). Although a disease-predisposing role has been demonstrated for *NPSR1* in several studies and in different populations, there has been only partial overlap of markers in previous investigations, and associations have been mainly accounted for by haplotypes tagged by single nucleotide polymorphisms (SNPs) with less distinct individual effect on disease risk.[11], [12], [14]–[27] *NPSR1* true causative variants remain to be identified, together with their specific effects on *NPSR1* expression and/or function.

**Figure 1:**
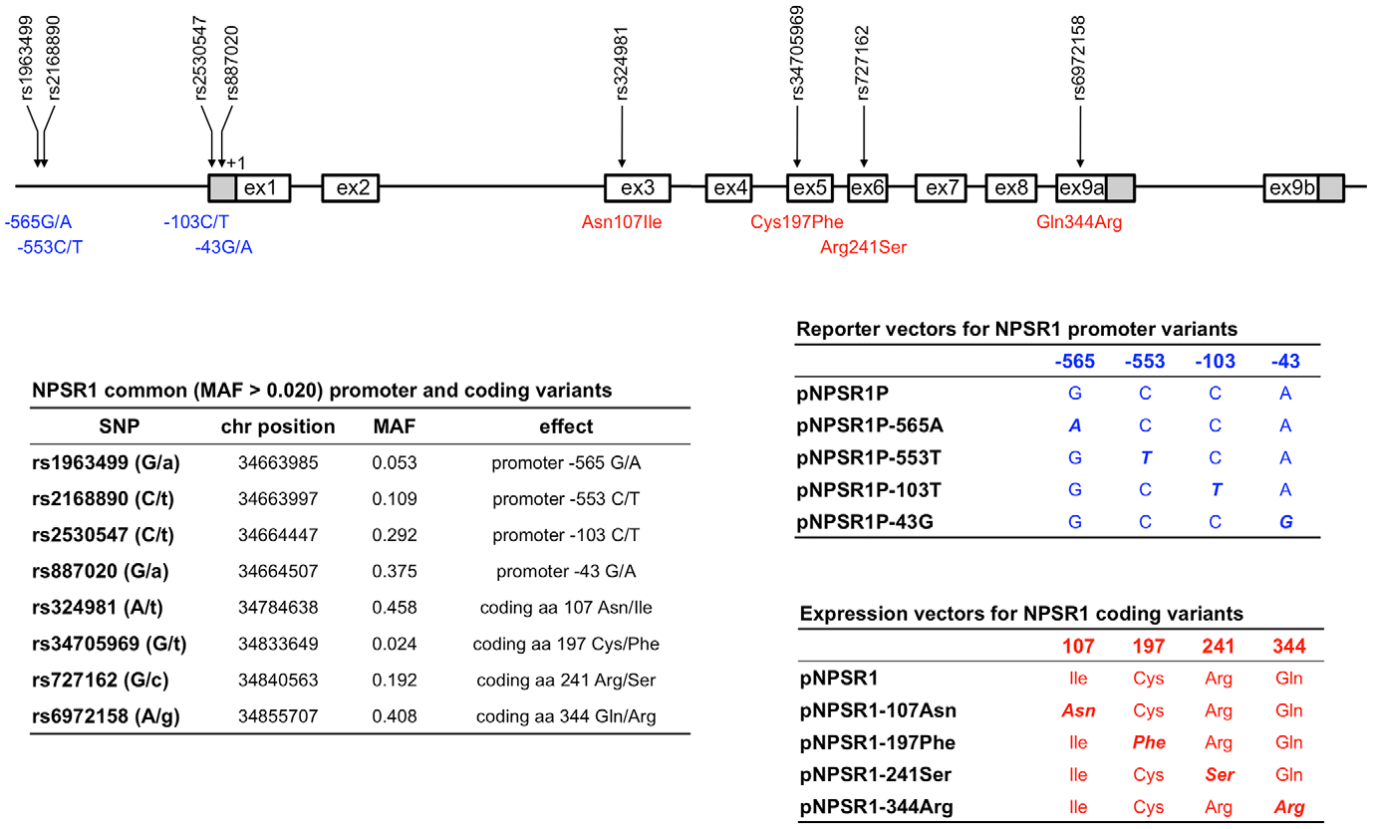
NPSR1 polymorphisms characterized in this study, and vector constructs used for their functional analysis. Top: NPSR1 common (minor allele frequency [MAF] >0.02) promoter and coding SNPs and their location in the gene region. Left: Chromosomal position, MAF, and functional effect of NPSR1 SNPs (minor alleles in lower case). Right: Vectors used to functionally characterize NPSR1 promoter (top) and coding (bottom) SNPs. For coding polymorphisms, a Myc-tagged version of each construct has also been produced for the experiments of immunofluorescence and FACS analysis.

The *NPSR1* gene spans 220 kb of genomic DNA on chromosome 7p14, in a region where >1000 SNPs have been detected through sequencing of individuals of different ethnicity. While, in theory, each SNP is of potential functional relevance, best candidates to play a causative role may be initially sought in regulatory and coding regions of the gene. Hence, to increase the likelihood of identifying such variants, we sought to characterize common (minor allele frequency [MAF] ≥0.02, figure 1) *NPSR1* promoter and coding polymorphisms for their impact on, respectively, the transcriptional regulation of gene expression and the signalling properties of the corresponding receptor.

## Materials and Methods

### Ethics statement

Ethical approval was obtained from the following local ethics committees: Karolinska Insitutet, Stockholm, Sweden and Örebro University Hospital, Örebro Sweden (for the genetic analyses carried out on IBD patients and controls); Amedeo Avogadro University, Novara, Italy (for the analysis of gene expression in peripheral blood). Written informed consent was obtained from Swedish IBD patients recruited at the Karolinska Institutet and Örebro University Hospital, and from healthy volunteers recruited at the Amedeo Avogadro University. Verbal informed consent was obtained from healthy blood donors recruited at the Blood Bank of the Örebro University Hospital. At arrival at the Blood Centre, blood donors were provided with written information, verbally informed about the study, and given the possibility to ask questions about the study. If a blood donor verbally approved to participate in the study, a blood sample was collected for the genetic analyses. This procedure was approved by the local ethical committee of the Örebro University Hospital, only age and sex of the blood donors were recorded and there is no way to trace back identities of the included individuals. Furthermore, the ethical committee approved that the consent was not recorded in the medical notes, since the enrolment was anonymous.

### Human material

The genetic analysis of *NPSR1* functional polymorphisms was carried out on 1525 IBD patients and 705 controls, from a large cohort of Swedish individuals described in detail in previous publications.[11], [28]–[32] Briefly, 659 Crohn's disease (CD) patients (aged 48.9±15.1 SD, 52.6% males), 866 ulcerative colitis (UC) patients (aged 52.1±16.3 SD, 56.7% males) and 705 healthy controls (aged 44.8±14.6 SD, 56.3% males) were recruited at the Karolinska University Hospital, Stockholm, and at the Örebro University Hospital, Örebro, Sweden, upon their written informed consent to participate in the study. Diagnosis of IBD (CD or UC) was based on standard clinical, endoscopic, radiologic and histologic criteria. Control individuals were healthy blood donors free of inflammatory disease, who provided verbal informed consent to be anonymously enrolled in the study.

For the correlation of NPSR1 mRNA expression with rs2530547 (−103) genotype, peripheral blood was obtained from 42 healthy volunteers (all white Caucasians, aged 30.2±6.6 SD, 40% males), recruited at Amedeo Avogadro University, Novara, Italy, upon written informed consent.

### Cloning and mutagenesis

*NPSR1* promoter was amplified from genomic DNA with primers CTCGAGGTCCTAGGTAGAAGCTGAGGT (forward) and AAGCTTGGCTCAGGCGGGGTTGAG (reverse), and cloned into pGL3-Basic Firefly Luciferase reporter vector (Promega, Madison, WI, USA) by using XhoI and HindIII restriction enzymes. NPSR1 expression vectors (full-length cDNA and N-terminal Myc-tagged full-length cDNA) have been previously described.[13] Allelic variants of *NPSR1* promoter and cDNA vectors were produced by site-directed mutagenesis using the QuickChange II Site Directed Mutagenesis kit (Stratagene, La Jolla, CA, USA). The entire inserts of all clones were sequence-verified. The specificity of each clone is reported in figure 1.

### Cell culture and transfection

HEK293 (ATCC CRL-1573) kidney epithelial and Colo205 (ATCC CCL-222) colon carcinoma cell lines were cultured in Minimal Essential Medium (MEM from Invitrogen, Carlsbad, CA, USA) and RPMI 1640 (Gibco BRL, Gaithersburg, MD, USA), respectively, supplemented with 10% foetal calf serum and 1% penicillin/streptomycin. Transfections were carried out with FUGENE 6 and FUGENE 6 HD (Roche Diagnostics, Basel, CH) respectively for HEK293 and Colo205, with 05–1** µ**g of total DNA according to manufacturer's instructions.

### Luciferase assays

For the study of *NPSR1* promoter SNPs, Colo205 cells were co-transfected with 900 ng of *NPSR1* promoter allelic constructs and 100 ng of pRL-TK Renilla Luciferase reporter vector (Promega, Madison, WI, USA), and total cell lysates were prepared 24 h post transfection. For pharmacokinetic studies, HEK293 cells were co-transfected with 400 ng of NPSR1 cDNA expression vectors specific for different coding variants and 50 ng each of cAMP response element (CRE) Firefly Luciferase reporter vector and CMV Renilla Luciferase reporter plasmid (purchased as pre-formulated mix from SABiosciences, Frederick, MD, USA). The day after transfection cells were stimulated for 6 h with NPS (MedProbe, Oslo, Norway) at various concentrations (ranging 10 pM – 100** µ**M) before preparation of total cell lysates. In all reporter assays, Luciferase activity was calculated using the Dual-Luciferase Reporter Assay System (Promega) according to manufacturer's instructions, and a Microplate Reader Infinite 200 (Tecan, Männedorf, CH). Experiments were done in triplicate, and Firefly Luciferase activity expressed in arbitrary units relative to the control vector, after normalization for transfection efficiency based on values obtained for Renilla Luciferase.

### Immunofluorescence

HEK293 cells were transfected with N-terminal Myc-tagged NPSR1 cDNA expression vectors corresponding to different coding variants. Twenty-four hours post transfection cells were fixed by incubation in 4% paraformaldehyde (in PBS) for 8–10 min at room temperature. The coverslips were blocked and primary rabbit anti-Myc tag antibody (Abcam, Cambridge, UK) was diluted in blocking buffer and incubated with the cells for 1–3 h. For the secondary antibody staining, Texas Red conjugated donkey anti-rabbit serum (Jackson Immunoresearch, West Grove, PA, USA) was diluted in blocking buffer containing 05 mg/ml Hoechst 33258 (Invitrogen) for identification of cell nuclei. Washed coverslips were then mounted in vinol mounting medium and images were captured using a Leitz DM RB fluorescent microscope Images were processed and compiled using Adobe Photoshop.

### Fluorescence-activated cell sorting (FACS) analysis

HEK293 cells were co-transfected with N-terminal Myc-tagged NPSR1 cDNA expression vectors corresponding to different coding variants and the pmaxGFP expression vector carrying green fluorescent protein cDNA (Amaxa, Cologne, Germany). Twenty-four h post transfection cells were treated with EDTA 0.5 M and then stained with anti-Myc primary antibody (Abcam) followed by Alexa Fluor 647 goat anti-rabbit IgG secondary antibody (Invitrogen). The analysis was performed on GFP positive cells.

### Real-Time PCR analysis

Total RNA was extracted from peripheral blood with commercially available kits (Qiagen, Hilden, Germany), and cDNA synthesized from 1** µ**g of RNA with SuperScript™ III First-Strand Synthesis SuperMix for qRT-PCR Kit (Invitrogen) according to manufacturer's instructions. *NPSR1* messenger RNA (mRNA) expression levels were measured by performing quantitative real-time PCR in an ABI Prism 7500 Fast Sequence Detection System (Applied Biosystems, Foster City, CA, USA) with SYBRGreen mix according to the manufacturer's instructions. Real-time PCR reactions were performed in triplicate on each sample with primers CCCCCTCATCTACTGTGTCTTCA (forward) and TCTCTCCCGGAACGTCATTCT (reverse) for *NPSR1*, and CCACATCGCTCAGACACCAT (forward) and GCGCCCAATACGACCAAAT (reverse) for glyceraldehyde 3-phosphate dehydrogenase (GAPDH), which was used as internal endogenous control. After normalization to GAPDH, NPSR1 mRNA expression levels in each sample were determined by the comparative C_T_ method of relative quantification, and expressed in arbitrary units relative to a randomly chosen reference sample.

### Microarray sample preparation and hybridizations

HEK293 cells were transfected with NPSR1 expression vectors specific for each coding variant and with an empty (control) vector, all in duplicate. Twenty-four h after transfection cells were stimulated with 1** µ**M NPS (final concentration) for 6 h and total RNA was purified with standard kits (Qiagen). RNA concentration and quality were measured with Agilent 2100 Bioanalyzer (Agilent Technologies, DE, USA), and a total of 8 µg of RNA from each sample was used for target cDNA synthesis according to the Affymetrix protocol. A total of 12 hybridizations (corresponding to duplicate transfections for 1 control vector and 5 NPSR1 plasmids) were performed on HGU133plus2 arrays (Affymetrix, Santa Clara, CA, USA), which were scanned with a GeneChip Scanner 3000 (Affymetrix) according to manufacturer's instructions and protocols for gene expression technology.

### Genotyping

Genomic DNA was isolated from peripheral blood with commercially available kits (Qiagen). The SNPs rs2530547 (−103), rs324981 (Ile107Asn), rs34705969 (Cys197Phe), and rs727162 (Arg241Ser) were genotyped in an ABI Prism 7500 Fast Sequence Detection System (Applied Biosystems), respectively with TaqMan® SNP Genotyping Assays C___2959938_10, C___2959781_10, C__58876042_10 and C___2277753_10, according to manufacturer's instructions.

### Molecular modelling

The molecular model of NPSR1 was created using the FUGUE Protein Modelling Server.[33] The crystal structure of the G protein-coupled receptor bovine rhodopsin (pdb identity code 1U19) was used as a template for the modelling.[34] Figures were created using the program PYMOL (http://www.pymol.org).

### Bioinformatics and statistical analyses

In silico prediction of the *NPSR1* promoter was obtained with the ARTS software (http://www.fml.tuebingen.mpg.de/raetsch/projects/arts). The TRANSFAC database was screened with MatInspector 4.3 (http://www.genomatix.de)to search for transcription factors (TFs) potentially binding to *NPSR1* predicted promoter. The effect of the SNP rs2530547 (−103) on TF binding was evaluated with Match 1.0 (http://www.gene-regulation.com) using a matrix similarity cut-off value of 0.8, while potential variation in kinase-specific phosphorylation at NPSR1 residue 241 (SNP rs727162) was predicted with NetPhosK (http://www.cbs.dtu.dk/services/NetPhosK).

For microarray analysis, the quality of the chips was assessed using the guidelines and the benchmarks from Affymetrix (http://www.affymterix.com). The software package Simpleaffy V 2.20.0 (http://www.bioconductor.org) from the Bioconductor Repository was used in order to estimate background, scale factors, percentage of present probesets, and GAPDH and beta-Actin expression levels. The array passed all standard quality control checks. Normalization of the microarray was done using the package Affy 1.22 from the Bioconductor Repository. We used only Perfect Match probes, invariant set, no background subtraction and the Li-Wong summary method.[35] The differential expression of genes/probesets was assessed using the hierarchical fitting method implemented in the software package LIMMA 2.18.1 (http://www.bioconductor.org) with Benjamini & Hochberg correction for multiple tests. We used a threshold p-value of 0.05 (after correction) to record a gene/probeset as differentially expressed, and only genes with fold changes >2 were taken into account, according to Affymetrix guidelines. Enrichment analysis of GO (Gene Ontology) terms was performed using the package GOSTATS 2.10.0 (http://www.bioconductor.org). Microarray data in compliance with the MIAME guidelines have been deposited in the ArrayExpress database (http://www.ebi.ac.uk/arrayexpress), with accession number E-MEXP-3226).

In the genetic analysis of *NPSR1* functional SNPs, associations between haplotypes and disease were tested using the algorithm haplocc from the R package haplo.stats 1.4.4 (http://www.r-project.org).[36] Haplotypes were reconstructed using an expectation maximization algorithm with progressive loci insertion. A generic log-additive model was used to test allelic effects for single markers, a common (allele frequency >0.10) haplotype was randomly chosen as baseline for OR calculations, and P values were estimated by a chi square test over the deviance of the regression. The Bonferroni correction method was adopted to take into account the number of tested phenotypes (IBD, UC and CD), and significance was set to P<0.017 (α = 0.05/3).

A Mann-Whitney U test was used for the statistical analysis of luciferase reporter assays performed with *NPSR1* promoter variants, and for the analysis of *NPSR1* expression in blood from healthy volunteers stratified according to genotype at the SNP rs2530547 (−103). To evaluate the significance of allele-specific differences (among *NPSR1* coding variants) in the magnitude of induction of differential gene expression following NPS stimulation, a Wilcoxon matched-pairs signed-ranks test was performed on genes showing fold change >4 in at least one transfection.

## Results

### Functional analysis of *NPSR1* promoter polymorphisms

Bioinformatic analysis of the region upstream of *NPSR1* coding sequence resulted in the prediction of a unique promoter spanning 595 bp 5′ of the translation initiation codon (ATG). Several transcription factors known to modulate neurological functions and inflammatory responses were predicted to bind to this region. Among these, OCT1, Pdx1, NEUROD1, MEIS1, Ap1 and Myb have been implicated in the regulation of expression of other neuropeptides such as the neuropeptides Y and FF and the vasoactive intestinal peptide (VIP). These results are graphically depicted in figure S1. To verify whether the identified region contains elements promoting transcription, we cloned the corresponding genomic DNA fragment into a luciferase-reporter vector, and used this construct to transiently transfect Colo205 cells, which are known to endogenously express *NPSR1* mRNA[4]. As shown in figure S1, this plasmid gave rise to a 4-fold increase in luciferase activity compared to the empty luciferase-reporter vector, supporting the results obtained from the bioinformatic prediction.

**Figure 2:**
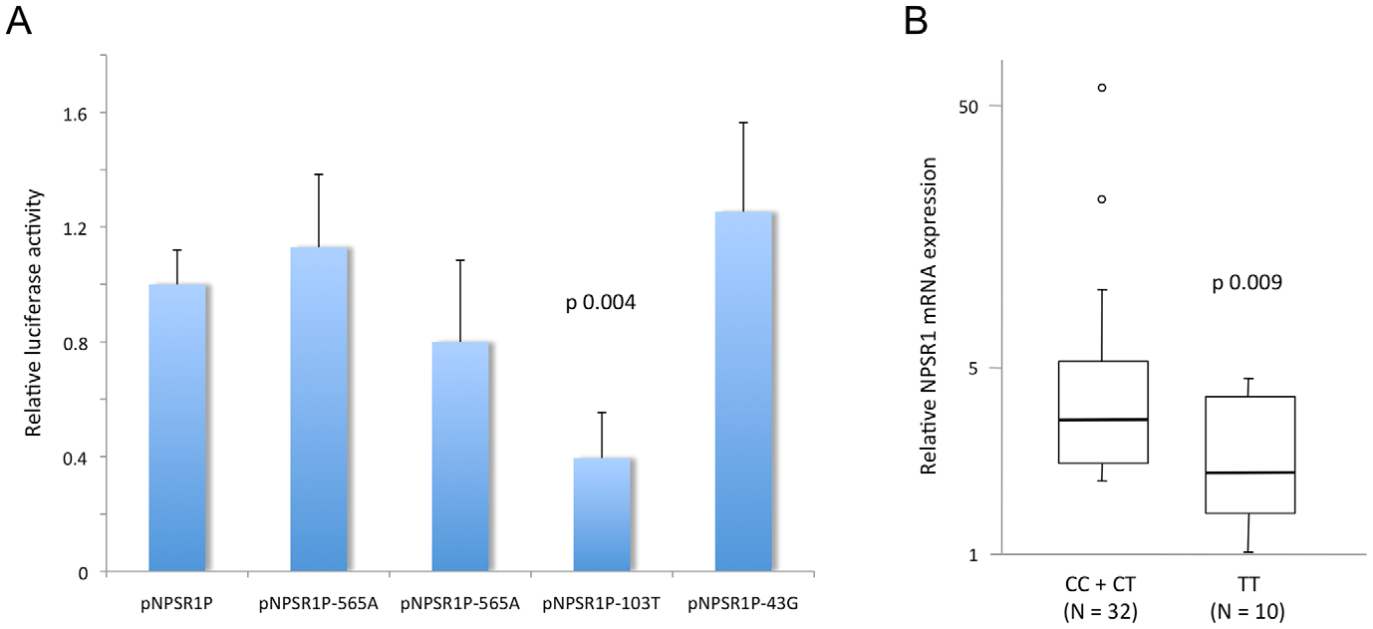
Effect of NPSR1 promoter polymorphism on gene transcription. A) Colo205 cells were transiently transfected with NPSR1 promoter constructs as indicated, in triplicate experiments. Twenty-four hours after transfection luciferase activity was measured and expressed as fold changes (+ SD) relative to the reference construct pNPSR1P. The substitution of a cytosine (C) with a thymidine (T) at position −103 (SNP rs2530547) resulted in significantly lower levels of luciferase activity (pNPSR1P-103T vs pNPSR1P, Mann-Whitney U test P = 0.004). B) Blood levels of NPSR1 mRNA were detected with TaqMan NPSR1-specific Real-Time PCR assay in 42 healthy volunteers, and expressed in arbitrary units according to genotype at position -103 of NPSR1 promoter (SNP rs2530547). Homozygosity for the T allele at this locus correlated with lower levels of NPSR1 mRNA (Mann-Whitney U test P = 0.009).

Four common SNPs (rs1963499, rs2168890, rs2530547 and rs887020) map within the *NPSR1* promoter, at positions −565, −553, −103 and −43, respectively, from the ATG start codon (figure 1 and figure S1). To assess their potential effect on *NPSR1* transcription, we generated corresponding variants of the *NPSR1* promoter plasmid (figure 1), and tested them in luciferase assays performed with transient transfections of Colo205 cells. As shown in figure 2A, while no relevant difference was detected for other SNPs, substitution of a cytosine (C) with a thymidine (T) at the −103 SNP site resulted in a significant reduction (62%) of luciferase activity. To confirm and further extend this result, we studied NPSR1 mRNA expression in peripheral blood from 42 healthy individuals with known rs2530547 genotype. As shown in figure 2B, this analysis also indicated that this SNP may influence transcriptional activity at the *NPSR1* locus, since −103 TT homozygosity was significantly associated with reduced levels of *NPSR1* mRNA expression. Bioinformatic analysis of the DNA sequence surrounding SNP rs2530547 predicted the −103T allele to disrupt a consensus sequence for the binding of ELK1 (Ets domain-containing protein Elk-1), a DNA binding protein of the ternary complex factor (TCF) subgroup of Ets transcription factors.

### Functional analysis of *NPSR1* coding polymorphisms

Four coding polymorphisms occur in the *NPSR1* gene with MAF>0.02, namely rs324981 (Ile107Asn), rs34705969 (Cys197Phe), rs727162 (Arg241Ser) and rs6972158 (Gln344Arg) (figure 1). To test their potential effect on receptor function, we used site-directed mutagenesis to generate NPSR1 expression vectors encoding receptor variants that differ at the corresponding polymorphic amino acid residues (namely 107Asn, 197Phe, 241Ser and 344Arg; figure 1). Because of the lack of suitable NPSR1-specific antibodies, we also produced Myc-tagged NPSR1 coding variants, which were uniquely used in receptor expression studies (although they also displayed NPS-induced signalling capacities comparable to their untagged equivalents, data not shown).

**Figure 3:**
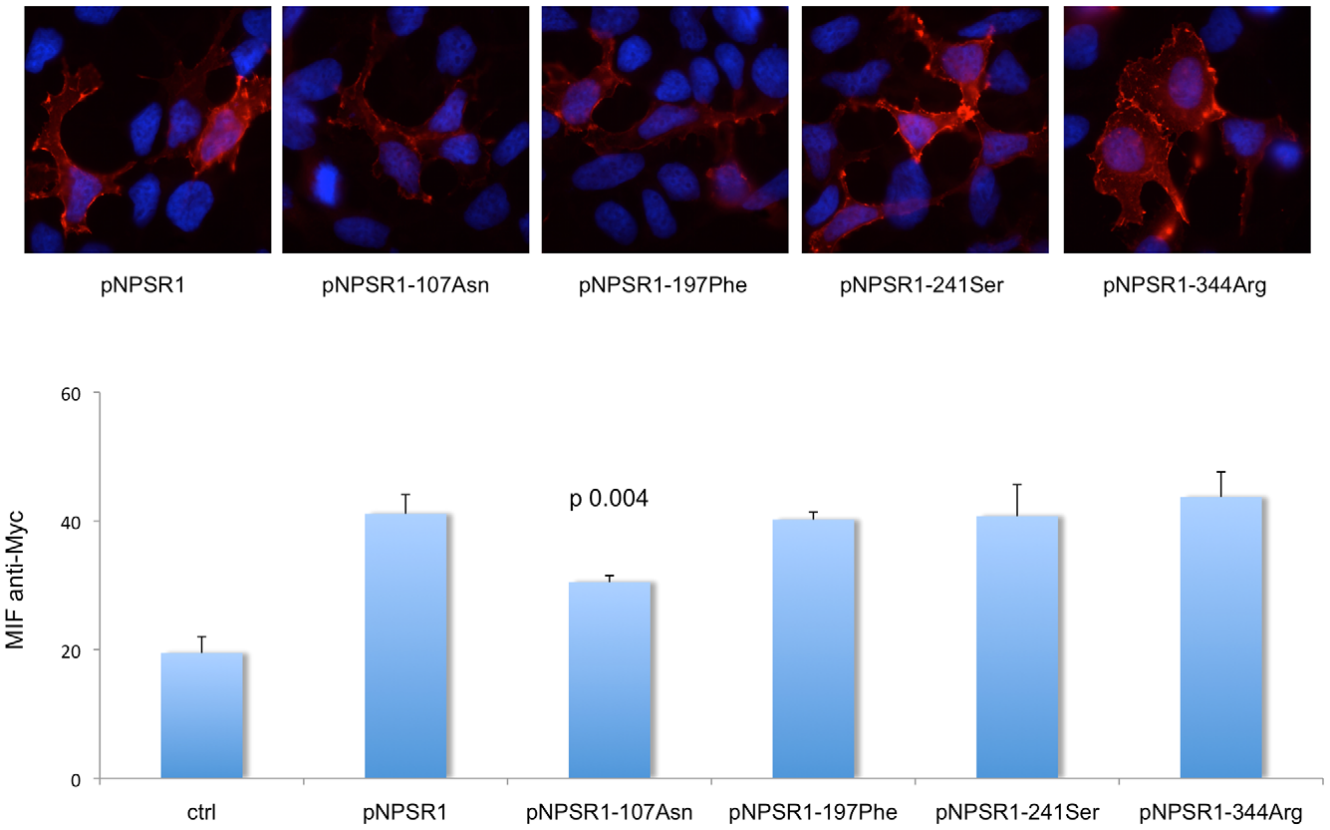
Membrane expression of NPSR1 coding variants. Top: HEK293 cells were transiently transfected with Myc-tagged NPSR1 expression vectors corresponding to different coding variants as indicated, and stained with anti-Myc antibody (red) and Hoechst 33258 (blue). Bottom: FACS analysis of cell surface expression of NPSR1 coding variants HEK293 cells were co-transfected with GFP and either an empty vector (ctrl) or vectors carrying Myc-tagged cDNAs for NPSR1 and all coding variants as indicated. After gating for GFP, anti-Myc mean fluorescence intensity (MIF) was measured for each sample, and the results obtained in three independent experiments are reported in the graph. In these conditions, the expression of the pNPSR1-107Asn variant was significantly lower (t test) than that of pNPSR1, taken as reference for this series of experiments.

We first tested whether amino acid changes corresponding to *NPSR1* alleles at coding SNPs have an effect on receptor expression. HEK293 epithelial cells were transiently transfected with Myc-tagged NPSR1 expression vectors and analyzed in immunofluorescence (IF). As shown in figure 3, no coding polymorphism disrupted NPSR1 expression on the cell membrane, since IF detection with an anti-Myc antibody gave rise to positive surface staining for all NPSR1 variants. To quantify their relative level of expression on the membrane, we then performed FACS experiments, and measured mean fluorescence intensity (MFI) in HEK293 cells transiently transfected with Myc-tagged NPSR1 coding variants. In these experiments, and as previously reported also by others,[37] the 107Asn clone resulted in a significantly lower level of NPSR1 membrane expression, but no relevant differences could be detected for other coding variants (figure 3).

A whole-transcriptome analysis was carried out to evaluate the potential impact of *NPSR1* coding SNPs on receptor signalling properties. HEK293 cells transiently transfected with different NPSR1 coding variants were stimulated with NPS for 6 h, and changes in gene expression downstream of NPS-NPSR1 signalling were quantified with Affymetrix GeneChip® Human Genome U133 Plus 2.0 arrays. As previously shown in HEK293 cells stably expressing the receptor,[10] analysis of microarray data based on Gene Ontology (GO) showed that NPSR1 signalling generally induces gene expression and transcriptional activation linked to nucleic acid metabolism, inflammatory responses and responses to stress or external stimuli (figure S2). We then compared the transcriptome profiles of different NPSR1 coding variants (available in full as table S1). As shown in Table 1, although no variant-specific pattern of gene expression was detected, the magnitude of NPS-induced transcriptional changes was markedly different for different NPSR1 coding variants. The most dramatic effect was observed for the substitution of a Cys with Phe in position 197 (pNPSR1-197Phe construct), which resulted in a loss-of-function pattern of gene expression, and virtually absent transcriptional induction of downstream genes (pNPSR1-197Phe:pNPSR1 mean fold change ratio = 0.26±0.18 SD, P<1.681×10^−10^). In addition, pNPSR1-107Asn and pNPSR1-241Ser coding constructs both gave rise to weaker changes in gene expression compared to the reference pNPSR1 receptor vector (pNPSR1-107Asn:pNPSR1 mean fold change ratio = 0.75±0.16 SD, P = 1.681×10^−10^, pNPSR1-241Ser:pNPSR1 mean fold change ratio = 0.93±0.08 SD, P = 3.473×10^−6^). In contrast, The 344Arg change had no significant effect on receptor signalling properties (pNPSR1-344Arg:pNPSR1 mean fold change ratio = 0.98±0.14 SD, P = 0.399).

**Table 1:** Genes differentially expressed upon NPS stimulation.

Affy probe	Gene	pNPSR1	pNPSR1	pNPSR1	pNPSR1	pNPSR1
			107Asn	197Phe	241Ser	344Arg
204637_at	CGA	+62.77	+36.23	−1.01	+46.55	+70.01
202859_x_at	IL8 a	+32.43	+16.34	−1.01	+30.47	+34.54
211143_x_at	NR4A1 a	+25.97	+18.04	+1.15	+22.35	+28.22
209774_x_at	CXCL2	+17.94	+9.73	+1.08	+17.09	+15.99
205476_at	CCL20	+16.18	+4.18	+1.00	+15.55	+20.22
206552_s_at	TAC1	+15.73	+12.06	−1.17	+12.77	+16.32
36711_at	MAFF a	+14.94	+12.22	+1.43	+15.30	+13.90
227099_s_at	LOC387763	+14.67	+10.59	+1.00	+14.56	+15.71
204621_s_at	NR4A2 a	+10.30	+8.24	−1.04	+9.47	+11.15
218541_s_at	C8orf4	+9.63	+6.35	+1.22	+9.88	+10.56
227404_s_at	EGR1 a	+8.55	+5.11	+1.22	+7.68	+7.75
207978_s_at	NR4A3 a	+8.47	+5.37	−1.03	+6.82	+8.75
207768_at	EGR4	+8.30	+3.55	+1.10	+9.14	+9.28
219716_at	APOL6	+7.22	+6.04	+3.61	+6.40	+4.86
202508_s_at	SNAP25	+7.15	+6.85	+1.95	+6.80	+6.24
1561434_at	C15orf45	+6.36	+5.94	+3.06	+6.42	+5.15
213321_at	BCKDHB	+6.30	+5.31	+2.83	+6.13	+4.49
204472_at	GEM	+6.19	+4.60	−1.06	+5.38	+6.04
209189_at	FOS	+5.73	+3.94	−1.01	+4.88	+5.80
202768_at	FOSB	+5.67	+3.50	+1.10	+4.71	+4.95
202014_at	PPP1R15A a	+5.49	+4.39	+1.75	+4.98	+4.47
210511_s_at	INHBA	+5.43	+3.66	−1.01	+4.69	+5.09
223774_at	SNHG12 a	+5.34	+5.30	+3.68	+5.69	+4.20
229709_at	ATP1B3	+5.29	+3.76	−1.10	+3.77	+5.96
206115_at	EGR3	+5.25	+3.10	+1.03	+5.52	+6.13
226164_x_at	RIMKLB a	+5.23	+5.22	+2.60	+5.32	+4.79
227140_at	NA	+5.09	+3.72	−1.04	+3.95	+5.04
202644_s_at	TNFAIP3 a	+4.96	+3.81	+1.80	+4.87	+4.87
202672_s_at	ATF3 a	+4.69	+4.08	+1.61	+4.65	+4.27
225557_at	CSRNP1	+4.55	+3.84	+1.32	+4.28	+4.18
204846_at	CP	+4.52	+4.38	+3.65	+4.66	+4.15
1559563_at	NA	+4.37	+3.74	+2.26	+3.90	+3.19
201473_at	JUNB	+4.34	+3.65	+1.72	+4.53	+4.52
228536_at	PRMT10	+4.33	+4.00	+1.26	+3.72	+3.93
209277_at	TFPI2 a	+4.33	+2.59	−1.07	+4.27	+5.57
210538_s_at	BIRC3	+4.31	+3.85	+2.16	+4.25	+4.41
218880_at	FOSL2	+4.14	+3.86	+1.25	+3.90	+3.42
226206_at	MAFK	+4.02	+3.43	+1.35	+3.97	+3.50
1559565_x_at	NA	+4.02	+3.37	+2.13	+3.67	+3.27
226099_at	ELL2	+3.93	+3.61	+1.09	+3.93	+4.29
205239_at	AREG	+3.91	+2.61	+1.03	+3.64	+4.73
1555571_at	IMMP2L	+3.81	+3.89	+2.71	+4.10	+3.37

NOTE: Differential expression is reported for each gene (Affymetrix probeset) and each NPSR1 variant in fold changes (fc) relative to cells transfected with an empty vector. Reported are genes for which a fc >4 has been detected in at least one transfection (+ denotes up-regulation, and - down-regulation).

a Highest fc values are reported for genes with significant results for multiple probesets.

**Figure 4:**
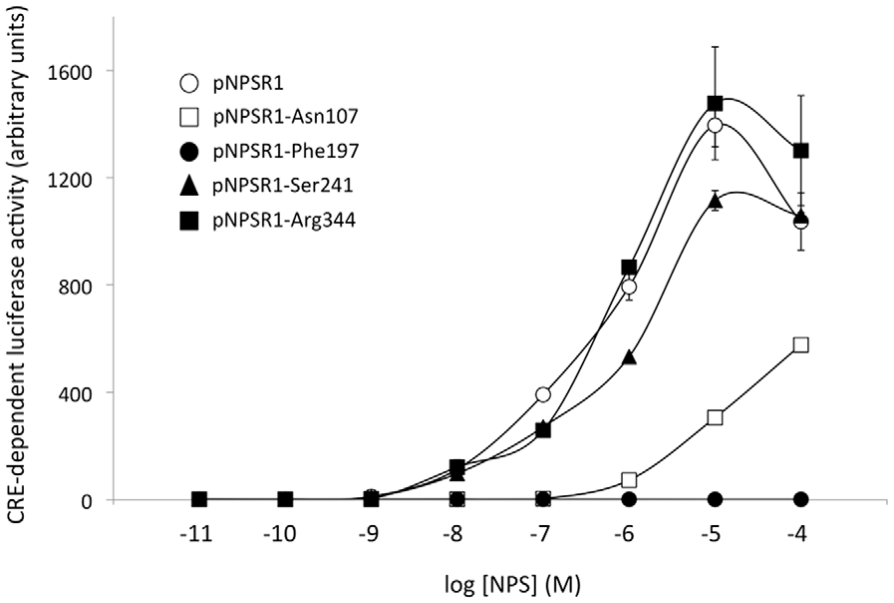
Pharmacological activity of NPSR1 coding variants. Dose-response curves of NPS stimulation, in HEK293 cells transiently co-transfected with a CRE-luc plasmid and NPSR1 coding variants in triplicate, as indicated. One representative experiment is reported, where CRE-driven luciferase activity is expressed in mean arbitrary units ± SEM.

To confirm and extend these functional results, we compared NPSR1 coding variants in another experimental setting, and assessed their dose-response to NPS in a luciferase assay dependent on the activation of a cAMP-response element (CRE). HEK293 cells were transiently co-transfected with different NPSR1 coding variants and a CRE-Luc plasmid, and luciferase activity measured 6 h after stimulation with serial dilutions of NPS. As shown in figure 4, most NPSR1 variants induced dose-dependent activation of luciferase activity, while pNPSR1-197Phe cells displayed complete lack of signalling induction at all tested concentrations of NPS. Also in these experimental conditions, NPSR1 coding variants showed quantitative differences similar to those observed in the transcriptome analysis, particularly at higher NPS concentrations, and the pNPSR1-241Ser and pNPSR1-107Asn transfected cells exhibited slightly and strongly reduced signalling properties, respectively, when compared to cells transfected with the reference pNPSR1 construct (figure 4).

**Figure 5:**
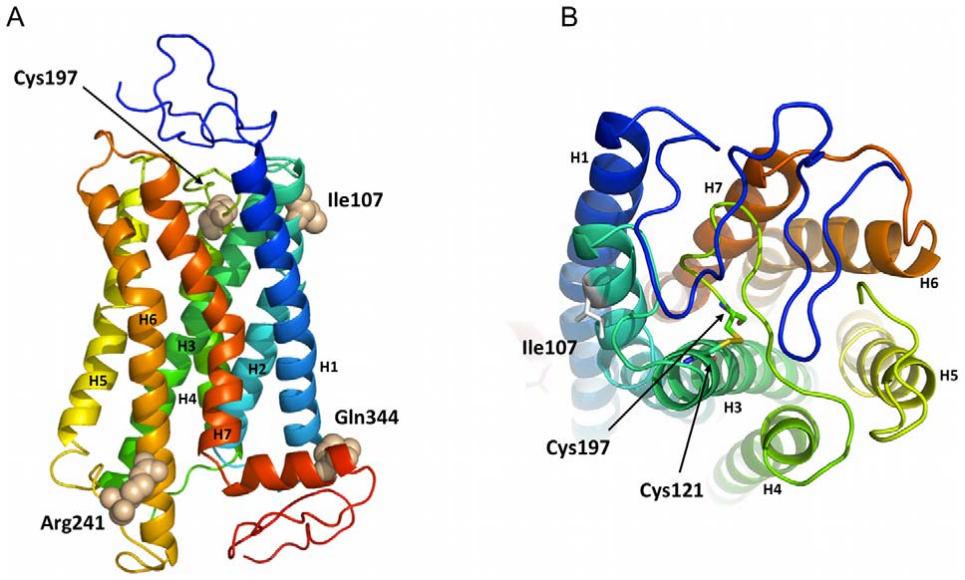
Molecular model of NPSR1. A) The molecular model of NSPR1 indicates that residues Ile107, Cys197 and Agr 241 are localized at each end of the transmembrane NSPR1 protein, while residue Cys197 is within the presumptive NPS-binding site. The model (side view) is oriented with the extracellular matrix and the cytosolic compartment at the top and at the bottom, respectively. Each of the seven helices that compose the NPSR1 transmembrane core is indicated with a different color and annotated with H. The side chains of the four different residues are displayed as spheres. B) View of the molecular model of NPSR1 seen from the extracyoplasmic domain. Residues Ile107, Cys197 and Cys121 are indicated as sticks. The side chains of Cys197 and Cys121 form a disulfide bridge within the NPS-binding site.

These functional experiments demonstrated that the three SNPs rs324981 (Ile107Asn), rs34705969 (Cys197Phe), and rs727162 (Arg241Ser) appear to affect NPSR1 signalling properties. The amino acid residues corresponding to these SNPs are all well conserved in NPSR1 across different species (figure S3). A molecular model of the NPSR1 protein was created based on its sequence similarity to the previously published crystal structure of the G protein-coupled receptor bovine rhodopsin (figure 5). Analysis of the model suggested that the residues isoleucine at position 107 (Ile107), cysteine at position 197 (Cys197), arginine at position 241 (Arg241), and glutamine at position 344 (Gln344) are localized on different sides of the transmembrane protein: Ile107 is at the extracellular end of the H2 helix, Cys197 is at the bottom of the second extracellular loop, Arg241 is positioned on a cytosolic domain that links helices H5 and H6, while Gln344 is localized on a short intra-cytosolic helical stretch at the end of the H8 helix (figure 5A). These results are in accordance with a recent study where the interactions of NSPR with several non-peptide antagonists were modelled, and where a similar localization for the residue107 on the terminal portion of the second transmembrane helix (H2) was reported.[38] Importantly, our model indicated that the side chain of Cys197 forms a disulfide bridge with the side chain of residue cysteine at position 121 (Cys121) within the core of the NPS binding domain (figure 5B). Disruption of the disulfide bridge through the substitution of Cys197 with a phenylalanine would probably have a profound effect on the overall conformation of the protein as well as the properties of the NPS ligand-binding pocket, which may impact ligand affinity and thus explain the observed loss-of-function phenotype of the 197Phe variant. While the structural relevance of residue 107 in antagonist binding has already been addressed in a previous study,[38] our model indicates that residue Arg241 is facing the intracellular compartment, and bioinformatic analysis of the surrounding sequence resulted in the prediction of consensus protein kinase A and C (PKA and PKC) phosphorylation sites overlapping with this position in the presence of a serine (figure S3). Hence, the NPSR1 Ile107Asn, Cys197Phe and Arg241Ser coding polymorphisms may act through different mechanisms to affect receptor function and signalling properties.

### Pilot study of *NPSR1* functional polymorphisms in IBD

Finally, in a preliminary analysis, we sought to test *NPSR1* functionally relevant SNPs for their potential association with disease. For this purpose, we genotyped the SNPs rs2530547 (−103), rs324981 (Ile107Asn), rs34705969 (Cys197Phe), and rs727162 (Arg241Ser) in 1525 IBD cases, comprising 659 CD patients and 866 UC patients, and 705 healthy controls from Sweden. Based on our results, we reasoned that a coherent analysis of *NPSR1* impact on disease risk needs to be performed by taking into account several functional polymorphisms at once, that is by testing *cis* allele combinations corresponding to “functional haplotypes”. However, as expected, the rs34705969 SNP had a very low minor allele frequency in our population (T = 0.023), where TT homozygous individuals were absent, and this polymorphism therefore was not included in the association tests. As shown in Table 2, where the results obtained from the analysis of the remaining SNPs are reported, we detected significant association with reduced risk of IBD for the functional haplotype rs2530547C-rs324981A-rs727162C (OR = 0.78; P = 0.0097), which is composed by alleles corresponding to increased NPSR1 mRNA expression (-103C) and weaker intracellular signalling (107Asn and 241Ser) at the respective polymorphic positions. The association signal was almost entirely due to a decreased frequency of the rs2530547C-rs324981A-rs727162C haplotype among UC patients (OR = 0.73; P = 0.0059), while a similar trend was observed among CD patients, although this did not reach statistical significance (OR = 0.83; P = 0.11). No other significant associations were detected in this relatively small sample set, including when the functional SNPs were individually tested (not shown).

**Table 2:** Association of NPSR1 functional haplotypes with IBD.

*rs2530547 (*−*103)*	*rs324981 (Ile107Asn)*	*rs727162 (Arg241Ser)*	*Ctrls (n = 705)*	*CD (n = 659)*				*UC (n = 866)*				*IBD (n = 1525)*			
			*f*	*f*	*P*	*OR*	*95%CI*	*f*	*P*	*OR*	*95%CI*	*f*	*P*	*OR*	*95%CI*
C	A	C	0.329	0.302	0.11	0.83	(0.63–1.10)	0.280	***0.0059***	0.73	(0.56–0.94)	0.289	***0.0097***	0.78	(0.62–0.98)
C	A	G	0.038	0.035	0.80	0.83	(0.47–1.48)	0.037	0.58	0.86	(0.50–1.46)	0.036	0.64	0.85	(0.53–1.37)
C	T	C	0.220	0.203	0.38	0.84	(0.60–1.17)	0.225	0.97	0.89	(0.65–1.21)	0.215	0.67	0.87	(0.66–1.15)
C	T	G	0.083	0.103	0.18	1.13	(0.78–1.63)	0.082	0.93	0.86	(0.60–1.24)	0.091	0.52	0.97	(0.71–1.34)
T	A	C	0.098	0.115	0.24	1.06	(0.69–1.62)	0.120	0.083	1.07	(0.72–1.59)	0.118	0.095	1.06	(0.74–1.52)
T	A	G	0.080	0.078	0.95	0.87	(0.59–1.29)	0.082	0.69	0.86	(0.60–1.24)	0.081	0.77	0.87	(0.63–1.20)
T	T	C	0.132	0.145	0.26	1.00	(baseline)	0.152	0.033	1.00	(baseline)	0.149	0.054	1.00	(baseline)
T	T	G	0.021	0.021	0.65	0.88	(0.34–2.28)	0.021	0.59	0.85	(0.34–2.11)	0.021	0.59	0.85	(0.38–1.92)

## Discussion

We report here a thorough characterization of *NPSR1* polymorphisms potentially affecting neuropeptide S receptor expression and function. Based on genotyping and sequencing data publicly available from the International HapMap Project, we selected and included in our study all *NPSR1* promoter and coding SNPs commonly found in the Caucasian population (minor allele frequency >0.02). Our data indicate that some of these polymorphisms impact both the regulation of *NPSR1* mRNA expression and the signalling properties of the corresponding receptor.

We identified a region upstream the *NPSR1* coding sequence that is able to drive transcription in the only cell line, Colo205, where *NPSR1* mRNA expression has been detected so far. Predicted binding sites for several transcription factors involved in neuronal and inflammatory responses map within this region, and we therefore propose this DNA sequence as the promoter, or at least one of the promoters, controlling transcription from the *NPSR1* locus. One of the *NPSR1* promoter SNPs, rs2530547 (−103), was associated with allelic effects on transcriptional activity, both in *in vitro* luciferase assays and in peripheral blood leukocytes from healthy volunteers, and may therefore be relevant to disease pathogenesis. Interestingly, the rs2530547 minor allele T is predicted to abolish a consensus binding site for the transcription factor ELK1 (Ets domain-containing protein Elk-1). ELK1 is a nuclear target for the mitogen-activated protein kinase (MAPK) signalling cascade, which is pro-inflammatory and is also induced upon NPS-NPSR1 signalling,[4],[39] and ELK1 thus represent an excellent candidate for further investigation on the regulation of NPSR1 expression.

Only the Ile107Asn coding SNP (rs324981) has been studied in the past for its functional impact on NPSR1 receptor properties.[4], [37], [40] Here, we characterized also the non-synonymous polymorphisms Cys197Phe (rs34705969), Arg241Ser (rs727162) and Gln344Arg (rs6972158) for their effect on NPSR1 in terms of membrane expression, transcriptome changes downstream of NPS signalling, pharmacological activity, and predicted structural properties of the receptor (rs34705969). Our data confirm previous findings on the Ile107Asn variation, with Ile associated with higher expression on the cell membrane, stronger intracellular cAMP signalling, and more potent induction of gene transcription changes compared to Asn. However, the results reported here also indicate that two other coding SNPs, namely Cys197Phe and Arg241Ser, have important functional effects on the NPSR1 receptor. Particularly impressive is the loss-of-function phenotype displayed by the substitution of Cys with Phe at residue 197 (Cys197Phe). Although retaining membrane expression, this receptor variant is not able to induce downstream gene expression changes or cAMP-mediated signalling upon NPS stimulation, even at high concentrations of ligand. As highlighted by our molecular model of NPSR1, this is possibly due to the disruption of a disulfide bridge between Cys192 and Cys197 when the latter is replaced by Phe. It should be noted that this cysteine bridge, also present in the crystal structure of bovine rhodopsin, is a highly conserved feature among GPCRs.[41] In the light of these findings, it will be important to test whether subjects carrying both copies of NPSR1 with Phe at residue 197 (SNP rs34705969 TT homozygotes) possess phenotypic features similar to those observed in *Npsr1* knockout mice, which are viable but show abnormal responses to stress and increased anxiety-like behaviours.[42] Though, the identification of such subjects may be difficult because of the rarity of this variant. Although not as dramatic as for SNPs at amino acids 107 and 197, important functional effects are also seen for the rs727162 coding polymorphism, which corresponds to an amino acid change from Arg to Ser at position 241 (Arg241Ser). In our experimental settings, the presence of a Ser at this position associates with diminished NPS-induced signalling, as seen both at the level of transcriptional changes in downstream target genes, and in the magnitude of the activation of cAMP-dependent luciferase expression. In our molecular model of NPSR1, the side chain of residue 241 faces the cytoplasm, and a consensus site for PKA and PKC phosphorylation is predicted only when a serine is present in this position. It is known that GPCR phosphorylation can induce receptor desensitization and attenuation of signalling, and these bioinformatic predictions are thus in accordance with the reduced activation of NPSR1 signalling for the 241Ser variant.

**Figure 6:**
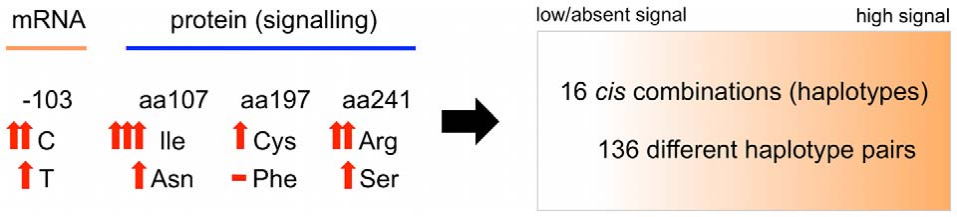
Functional variability in NPSR1 receptor signalling. Based on the results obtained in this study, allelic differences in mRNA expression and protein signalling capacity exist, respectively, at SNPs corresponding to promoter position −103 and amino acid residues 107, 197 and 241. This is graphically represented by vertical arrows in the figure, where more arrows indicate higher expression or stronger signalling, respectively, for promoter and coding SNPs. In theory, this results in 16 potential *cis* combinations of *NPSR1* functional alleles into haplotypes, and 136 different pairs of such haplotypes from 2 chromosomes, establishing a very large gradient of NPSR1 functional variation ranging from low/absent to very high signalling.

Although sometimes with contradictory results, numerous studies have associated *NPSR1* polymorphisms with human conditions such as asthma and related traits, IBD, functional gastrointestinal disorders, rheumatoid arthritis, and panic disorders.[11], [12], [14]–[27] However, neither the true causative variations, nor the functional mechanisms responsible for their effect on disease risk, have been conclusively identified in any of these studies, and most of the reported associations are with tag SNPs, intronic markers, or their haplotypic combinations. The large *NPSR1* gene spans 220 kb of DNA sequence on chromosome 7p, with more than 1000 SNPs and several LD blocks identified in different populations, and this has hampered the identification of risk alleles at this locus. The results reported here suggest that there are at least 4 functional SNPs in the *NPSR1* gene, and previous investigations may therefore need to be re-evaluated in the light of this finding. In particular, it seems correct to assume that testing associations with individual *NPSR1* SNPs may not be sufficiently informative, since a higher number of polymorphisms ultimately determine receptor properties and/or expression levels (figure 6). These polymorphisms likely exert different effects on disease risk depending on their respective combinations in *cis* and, since promoter and coding functional SNPs do not map within the same LD block in the *NPSR1* gene (figure S4), true risk combinations may need to be sought irrespective of the LD structure at the NPSR1 locus. Although not all NPSR1 haplotypes could be adequately tested because of low allelic frequencies, our preliminary analysis of “functional haplotypes” in a limited number of IBD patients and controls appears to support this hypothesis. First, we detected all possible combinations of alleles at the tested SNPs (2^3^ = 8 haplotypes), confirming that these polymorphisms are not in strong LD. Second, although modest, both the strength of the association (P = 0.0059) and the magnitude of the risk effect (OR = 0.73) observed for the haplotype rs2530547C-rs324981A-rs727162C in UC are comparable with those detected in much larger studies previously performed on several thousands individuals. The rs2530547C-rs324981A-rs727162C protective haplotype should correspond to a highly expressed (−103C) NPSR1 protein characterized by reduced signalling properties (107Asn and 241Ser), which is an intriguing observation requiring mechanistic explanation. This is unless the protective effect is mediated in heterozygosity with high-signalling NPSR1 receptor variants, a hypothesis we did not have enough statistical power to test.

With the limitation that only Caucasian common *NPSR1* variants have been functionally tested, this study takes us a step closer to understanding how genetic variability at the corresponding locus impact human disease, and pave the way to future studies on larger cohorts of cases and controls. Moreover, additional *NPSR1* rare variants have been recently discovered within the frame of the 1000 Genome Project Consortium,[43] and similar to the SNP rs34705969 (Cys197Phe) which shows dramatic functional effects, have never been tested for association in human diseases. Because of the pleiotropic nature of the NPS-NPSR1 system in immune, endocrine and neurological functions, it cannot be excluded that *NPSR1* functional polymorphisms may have different risk effects in different disorders.

Finally, the NPS-NPSR1 system holds great potential for therapeutic exploitation of several diseases when full information on its biological role is available, and the results reported here may also be of pharmacogenetic relevance. A number of NPS agonists and antagonists has already been identified,[44]–[48] and allele-specific potency of NPSR1 activation already shown for some NPS mutant peptides, depending on the residue present at the polymorphic sites Ile107Asn.[40] It will now be important to verify whether the differences observed here for some NPSR1 coding SNPs are also conserved when NPS agonists or antagonists are used instead of the natural ligand. If so, it is congruous to predict that a NPS agonist- or antagonist-based therapy would inevitably fail in homozygous carriers of a Phe at the Cys197Phe polymorphic site.

## Supporting Information

Figure S1 **Characterization of NPSR1 predicted promoter.** Left: ARTS-predicted NPSR1 promoter sequence, spanning nucleotides -595 to +1 from the translational start site (ATG). Transcription factors (TFs) predicted to bind to the promoter from MatInspector analysis are also reported, with their recognitions sites underlined. Among these, TFs involved in the modulation of inflammatory responses are shown in blue, while TFs known to regulate neurological functions are shown in green. Four common (MAF>0.02) SNPs mapping within the predicted promoter region are reported in red, together with the corresponding alleles at each site. Right: assessment of the functional activity of NPSR1 predicted promoter in luciferase reporter assays. Colo205 cells were transiently transfected with either a promoterless luciferase vector (pGL3-basic) or the same vector carrying NPSR1 promoter driving luciferase transcription (pNPSR1P). Results, which are representative of 3 independent experiments performed in duplicate, are expressed as fold induction, relative to the luciferase activity obtained for the control transfection (empty vector).
(TIF)

Figure S2 **Gene Ontology (GO) analysis of genes differentially expressed upon NPS/NPSR1 signaling.** Biological process annotations of the hits identified through microarray analysis of NPS-induced changes in gene expression, in HEK293 cells transiently transfected with NPSR1 expression plasmid vs cells transfected with empty vector.
(TIF)

Figure S3 **Characteristics of NPSR1 coding polymorphisms.** Left: alignment of NPSR1 coding SNPs 107 (rs324981), 197 (rs34705969), 241 (rs727162) and 344 (rs6972158) in different species. Only residue 344 shows some variability, while residues 107, 197 and 241 are conserved across all species. Right: Bioinformatic prediction of protein kinase A (PKA) and protein kinase C (PKC) phosphorylation sites in the region surrounding the coding SNP 241 (rs727162). Reported is the output of NetPhosK analysis when a Serine is present at the polymorphic residue 241. Letter size corresponds proportionally to the score values obtained from the analysis (PKA = A = 0.57; PKC = C = 0.61, at position 241). No phosphorylation is predicted to occur when an Arginine is present at residue 241 (only Serine (S), Threonine (T) and Tyrosine (Y) are substrate for PKA and PKC phosphorylation).
(TIF)

Figure S4 **Linkage disequilibrium (LD) structure of the NPSR1 gene region.** LD displayed by GOLD heatmap in the NPSR1 gene region (chr7:34662000–34887000), generated with Haploview on genotype data from the CEU (CEPH) population of European descent. NPSR1 exons (vertical blocks) and the functional SNPs identified in our study are reported on top of the LD plot.
(TIF)

Table S1 **NPS-inducd differential gene expression in cells transfected with the indicated NPSR1 variant vs cells transfected with an empty vector.** Genes/probesets with fold change >2 in at least one transfection are reported (- denotes downregulation). Fold change values <2 are reported only for comparison, and are highlighted in italics.
(PDF)
