# Supplementary figures and images for: Multiple Polymorphisms Affect Expression and Function of the Neuropeptide S Receptor (NPSR1)
Source: PLoS One. 2011 Dec 21;6(12):e29523. doi: 10.1371/journal.pone.0029523 (PMC3244468; doi:10.1371/journal.pone.0029523)

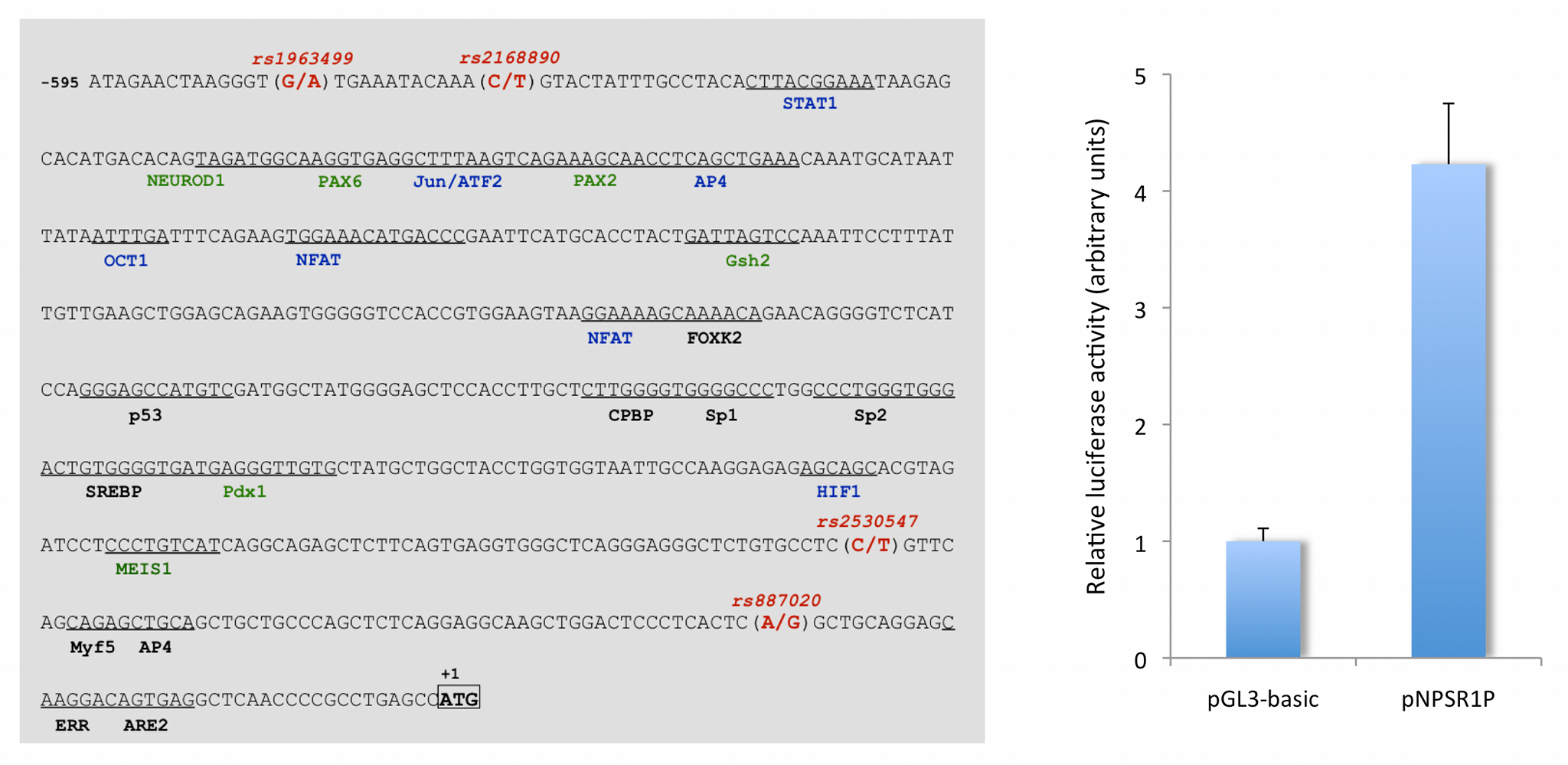

Supplement: Figure S1 — Characterization of NPSR1 predicted promoter. Left: ARTS-predicted NPSR1 promoter sequence, spanning nucleotides -595 to +1 from the translational start site (ATG). Transcription factors (TFs) predicted to bind to the promoter from MatInspector analysis are also reported, with their recognitions sites underlined. Among these, TFs involved in the modulation of inflammatory responses are shown in blue, while TFs known to regulate neurological functions are shown in green. Four common (MAF>0.02) SNPs mapping within the predicted promoter region are reported in red, together with the corresponding alleles at each site. Right: assessment of the functional activity of NPSR1 predicted promoter in luciferase reporter assays. Colo205 cells were transiently transfected with either a promoterless luciferase vector (pGL3-basic) or the same vector carrying NPSR1 promoter driving luciferase transcription (pNPSR1P). Results, which are representative of 3 independent experiments performed in duplicate, are expressed as fold induction, relative to the luciferase activity obtained for the control transfection (empty vector). (TIF) [file pone.0029523.s001.tif]

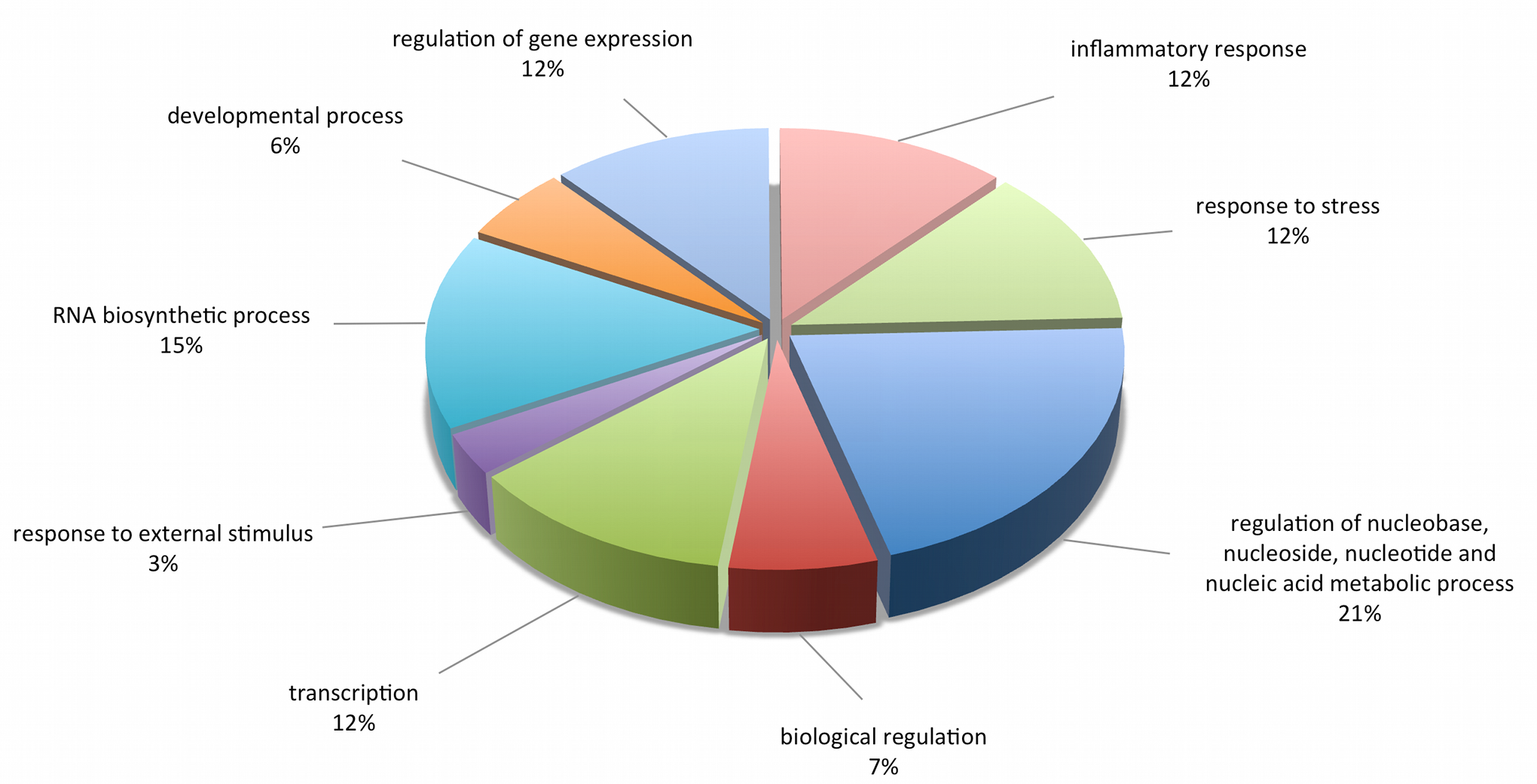

Supplement: Figure S2 — Gene Ontology (GO) analysis of genes differentially expressed upon NPS/NPSR1 signaling. Biological process annotations of the hits identified through microarray analysis of NPS-induced changes in gene expression, in HEK293 cells transiently transfected with NPSR1 expression plasmid vs cells transfected with empty vector. (TIF) [file pone.0029523.s002.tif]

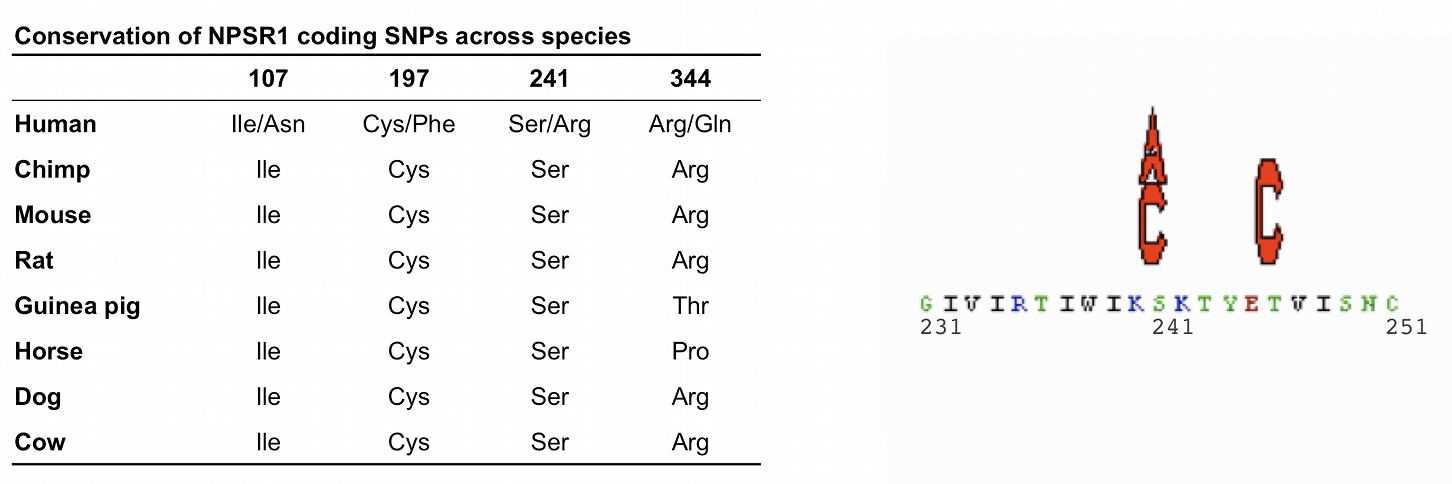

Supplement: Figure S3 — Characteristics of NPSR1 coding polymorphisms. Left: alignment of NPSR1 coding SNPs 107 (rs324981), 197 (rs34705969), 241 (rs727162) and 344 (rs6972158) in different species. Only residue 344 shows some variability, while residues 107, 197 and 241 are conserved across all species. Right: Bioinformatic prediction of protein kinase A (PKA) and protein kinase C (PKC) phosphorylation sites in the region surrounding the coding SNP 241 (rs727162). Reported is the output of NetPhosK analysis when a Serine is present at the polymorphic residue 241. Letter size corresponds proportionally to the score values obtained from the analysis (PKA = A = 0.57; PKC = C = 0.61, at position 241). No phosphorylation is predicted to occur when an Arginine is present at residue 241 (only Serine (S), Threonine (T) and Tyrosine (Y) are substrate for PKA and PKC phosphorylation). (TIF) [file pone.0029523.s003.tif]

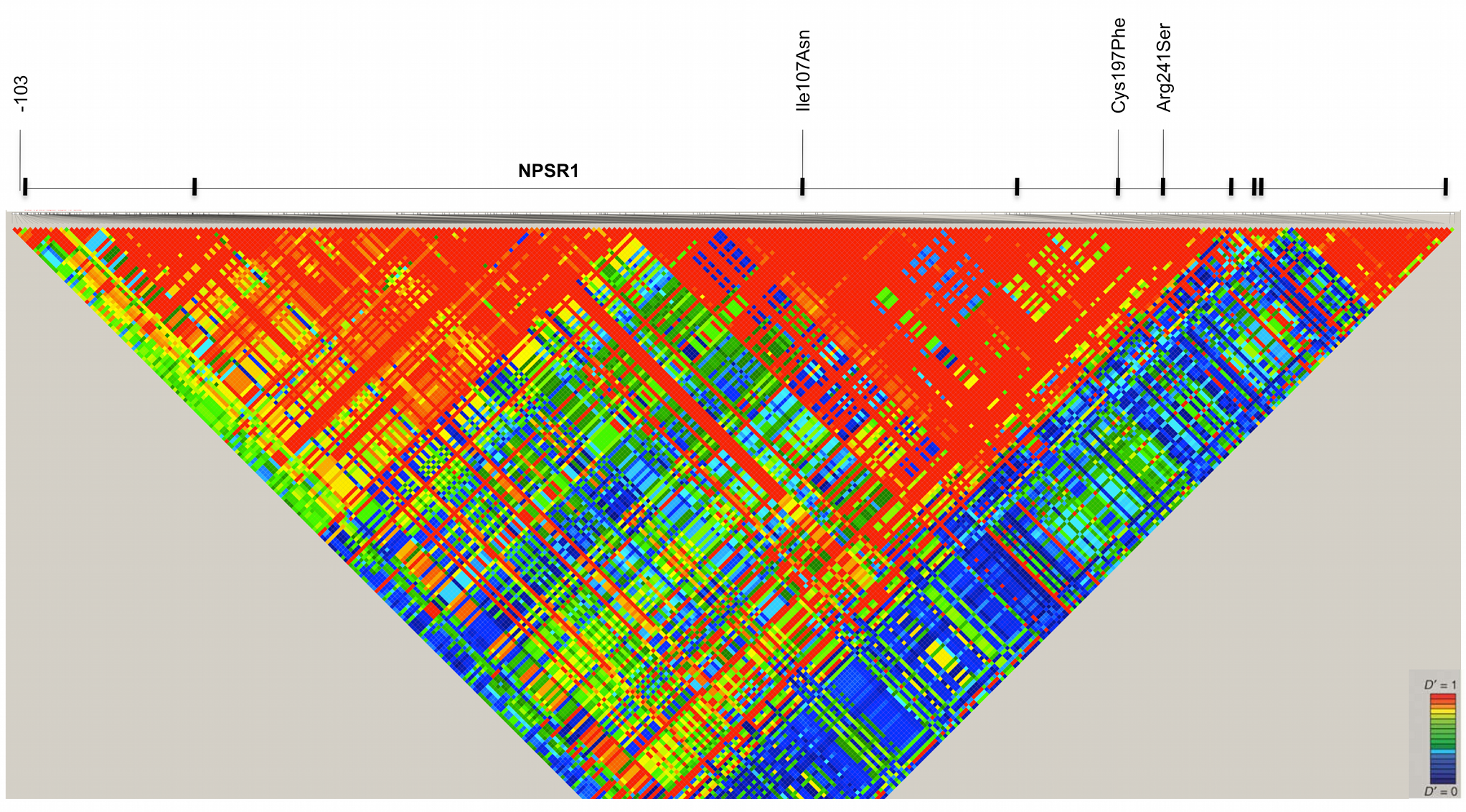

Supplement: Figure S4 — Linkage disequilibrium (LD) structure of the NPSR1 gene region. LD displayed by GOLD heatmap in the NPSR1 gene region (chr7:34662000–34887000), generated with Haploview on genotype data from the CEU (CEPH) population of European descent. NPSR1 exons (vertical blocks) and the functional SNPs identified in our study are reported on top of the LD plot. (TIF) [file pone.0029523.s004.tif]
