# Supplementary material for: Multiple Polymorphisms Affect Expression and Function of the Neuropeptide S Receptor (NPSR1)
Source: PLoS One. 2011 Dec 21;6(12):e29523. doi: 10.1371/journal.pone.0029523 (PMC3244468; doi:10.1371/journal.pone.0029523)
Supplement: Table S1 — NPS-inducd differential gene expression in cells transfected with the indicated NPSR1 variant vs cells transfected with an empty vector. Genes/probesets with fold change >2 in at least one transfection are reported (- denotes downregulation). Fold change values <2 are reported only for comparison, and are highlighted in italics. (PDF) [file pone.0029523.s005.pdf]

Table S1. NPS-inducd differential gene expression in cells transfected with the indicated NPSR1 variant vs cells transfected with an empty vector. Genes/probesets with fold change > 2 in at least one transfection are reported (- denotes downregulation). Fold change values < 2 are reported only for comparison, and are highlighted in *italics*.

		NPSR1		NPSR1-107Asn		NPSR1-197Phe		NPSR1-241Ser		NPSR1-344Arg	
**Affy probeset**	**Gene**	**fold change**	**p value**	**fold change**	**p value**	**fold change**	**p value**	**fold change**	**p value**	**fold change**	**p value**
**204637_at**	**CGA**	62.77	4.88E-13	36.23	2.00E-12	*-1.01*	*0.93028546*	46.55	9.68E-13	70.01	4.34E-13
**202859_x_at**	**IL8**	32.43	4.88E-13	16.34	2.00E-12	*-1.01*	*0.8548493*	30.47	6.93E-13	34.54	4.34E-13
**211143_x_at**	**NR4A1**	25.97	5.48E-12	18.04	1.54E-11	*1.15*	*0.087927*	22.35	8.22E-12	28.22	4.92E-12
**209774_x_at**	**CXCL2**	17.94	1.90E-12	9.73	1.11E-11	*1.08*	*0.18352002*	17.09	2.10E-12	15.99	3.30E-12
**205476_at**	**CCL20**	16.18	5.48E-12	4.18	9.03E-10	*1.00*	*0.94209248*	15.55	6.40E-12	20.22	3.42E-12
**206552_s_at**	**TAC1**	15.73	1.55E-11	12.06	3.60E-11	*-1.17*	*0.04571387*	12.77	3.19E-11	16.32	1.57E-11
**36711_at**	**MAFF**	14.94	2.82E-12	12.22	5.84E-12	*1.43*	*9.18E-05*	15.30	2.42E-12	13.90	3.42E-12
**227099_s_at**	**LOC387763**	14.67	1.38E-11	10.59	3.60E-11	*1.00*	*0.98860351*	14.56	1.33E-11	15.71	1.24E-11
**202340_x_at**	**NR4A1**	11.41	1.16E-09	7.51	4.83E-09	*1.12*	*0.32260783*	9.67	1.90E-09	11.65	1.27E-09
**211506_s_at**	**IL8**	11.02	2.44E-10	4.93	5.21E-09	*1.02*	*0.88229636*	10.21	3.11E-10	13.24	1.66E-10
**204621_s_at**	**NR4A2**	10.30	1.75E-09	8.24	4.00E-09	*-1.04*	*0.78243673*	9.47	2.27E-09	11.15	1.66E-09
**204622_x_at**	**NR4A2**	9.98	1.56E-10	8.10	2.89E-10	*1.00*	*0.99614853*	8.71	2.42E-10	9.22	2.16E-10
**216248_s_at**	**NR4A2**	9.92	1.02E-10	7.71	2.50E-10	*-1.09*	*0.29306749*	8.45	1.97E-10	9.20	1.66E-10
**218541_s_at**	**C8orf4**	9.63	3.90E-11	6.35	1.63E-10	*1.22*	*0.01304673*	9.88	3.28E-11	10.56	3.35E-11
**227404_s_at**	**EGR1**	8.55	2.44E-10	5.11	2.10E-09	*1.22*	*0.03138886*	7.68	3.70E-10	7.75	4.00E-10
**207978_s_at**	**NR4A3**	8.47	4.73E-09	5.37	2.71E-08	*-1.03*	*0.86147088*	6.82	1.08E-08	8.75	5.19E-09
**207768_at**	**EGR4**	8.30	2.44E-10	3.55	1.17E-08	*1.10*	*0.27652133*	9.14	2.04E-10	9.28	2.10E-10
**209959_at**	**NR4A3**	7.97	4.89E-11	6.19	1.24E-10	*1.03*	*0.68193313*	7.21	6.33E-11	7.58	7.21E-11
**205193_at**	**MAFF**	7.54	6.12E-11	6.26	1.24E-10	*1.20*	*0.01418803*	7.61	5.90E-11	7.04	1.00E-10
**219716_at**	**APOL6**	7.22	8.29E-08	6.04	1.45E-07	3.61	1.90E-05	6.40	1.28E-07	4.86	6.77E-07
**202508_s_at**	**SNAP25**	7.15	4.58E-10	6.85	5.76E-10	*1.95*	*1.42E-05*	6.80	5.53E-10	6.24	9.61E-10
**201694_s_at**	**EGR1**	6.96	2.39E-11	4.89	1.14E-10	*1.12*	*0.05141181*	6.35	3.28E-11	6.32	4.13E-11
**1561434_at**	**C15orf45**	6.36	7.60E-08	5.94	8.95E-08	3.06	3.02E-05	6.42	7.28E-08	5.15	2.71E-07
**213321_at**	**BCKDHB**	6.30	7.06E-08	5.31	1.26E-07	2.83	4.53E-05	6.13	7.73E-08	4.49	4.74E-07
**204472_at**	**GEM**	6.19	2.44E-10	4.60	1.13E-09	*-1.06*	*0.42925568*	5.38	5.23E-10	6.04	3.57E-10
**209189_at**	**FOS**	5.73	2.98E-09	3.94	1.71E-08	*-1.01*	*0.91418939*	4.88	6.06E-09	5.80	3.45E-09
**202768_at**	**FOSB**	5.67	3.49E-09	3.50	3.73E-08	*1.10*	*0.32723654*	4.71	7.85E-09	4.95	8.07E-09
**202014_at**	**PPP1R15A**	5.49	1.45E-09	4.39	4.67E-09	*1.75*	*5.66E-05*	4.98	2.27E-09	4.47	4.83E-09
**210511_s_at**	**INHBA**	5.43	2.44E-10	3.66	2.07E-09	*-1.01*	*0.93863083*	4.69	5.23E-10	5.09	3.99E-10
**223774_at**	**SNHG12**	5.34	8.25E-09	5.30	8.37E-09	3.68	8.94E-07	5.69	6.06E-09	4.20	3.47E-08
**229709_at**	**ATP1B3**	5.29	2.92E-07	3.76	1.58E-06	*-1.10*	*0.60888815*	3.77	1.81E-06	5.96	2.41E-07

		NPSR1		NPSR1-107Asn		NPSR1-197Phe		NPSR1-241Ser		NPSR1-344Arg	
**Affy probeset**	**Gene**	**fold change**	**p value**	**fold change**	**p value**	**fold change**	**p value**	**fold change**	**p value**	**fold change**	**p value**
**223773_s_at**	**SNHG12**	5.26	5.04E-11	5.25	5.47E-11	3.64	1.16E-08	5.50	3.94E-11	4.08	2.10E-10
**206115_at**	**EGR3**	5.25	1.43E-09	3.10	2.47E-08	1.03	0.78671753	5.52	1.19E-09	6.13	9.61E-10
**226164_x_at**	**RIMKLB**	5.23	2.44E-10	5.22	2.50E-10	2.60	3.65E-07	5.32	2.42E-10	4.79	4.28E-10
**227140_at**	**NA**	5.09	2.19E-08	3.72	9.84E-08	-1.04	0.79294217	3.95	7.78E-08	5.04	3.20E-08
**229344_x_at**	**RIMKLB**	5.06	8.98E-11	5.02	1.04E-10	2.59	1.31E-07	5.09	8.03E-11	4.64	1.66E-10
**202644_s_at**	**TNFAIP3**	4.96	1.19E-08	3.81	4.01E-08	1.80	0.00016854	4.87	1.24E-08	4.87	1.69E-08
**202643_s_at**	**TNFAIP3**	4.75	1.41E-08	3.21	1.07E-07	1.70	0.00037151	4.34	2.32E-08	4.84	1.79E-08
**202672_s_at**	**ATF3**	4.69	7.70E-10	4.08	1.65E-09	1.61	4.64E-05	4.65	7.96E-10	4.27	1.45E-09
**225557_at**	**CSRNP1**	4.55	4.66E-09	3.84	1.06E-08	1.32	0.00779544	4.28	6.06E-09	4.18	8.94E-09
**204846_at**	**CP**	4.52	4.74E-08	4.38	5.04E-08	3.65	1.95E-06	4.66	4.21E-08	4.15	1.03E-07
**1559563_at**	**NA**	4.37	1.33E-07	3.74	2.75E-07	2.26	9.60E-05	3.90	2.43E-07	3.19	1.27E-06
**201473_at**	**JUNB**	4.34	1.10E-09	3.65	2.92E-09	1.72	1.68E-05	4.53	8.43E-10	4.52	1.01E-09
**228536_at**	**PRMT10**	4.33	1.13E-08	4.00	1.52E-08	1.26	0.026361	3.72	2.43E-08	3.93	2.36E-08
**209277_at**	**TFPI2**	4.33	2.44E-10	2.59	6.25E-09	-1.07	0.27827563	4.27	2.57E-10	5.57	9.39E-11
**210538_s_at**	**BIRC3**	4.31	1.03E-07	3.85	1.65E-07	2.16	0.00010357	4.25	1.07E-07	4.41	1.26E-07
**37028_at**	**PPP1R15A**	4.22	1.22E-08	3.33	4.08E-08	1.54	0.00064119	3.86	2.01E-08	3.54	4.19E-08
**1554980_a_at**	**ATF3**	4.19	1.41E-07	3.25	5.83E-07	1.37	0.02712987	4.10	1.54E-07	3.81	3.40E-07
**218880_at**	**FOSL2**	4.14	5.91E-09	3.86	8.37E-09	1.25	0.01880008	3.90	7.85E-09	3.42	2.29E-08
**226206_at**	**MAFK**	4.02	5.96E-10	3.43	1.57E-09	1.35	0.00043323	3.97	5.94E-10	3.50	1.50E-09
**1559565_x_at**	**NA**	4.02	2.37E-07	3.37	6.07E-07	2.13	0.00018156	3.67	4.10E-07	3.27	1.25E-06
**209278_s_at**	**TFPI2**	3.95	1.21E-09	2.35	4.01E-08	-1.16	0.04216518	3.78	1.61E-09	4.88	4.57E-10
**226099_at**	**ELL2**	3.93	9.77E-07	3.61	1.35E-06	1.09	0.64372311	3.93	9.25E-07	4.29	8.23E-07
**1555379_at**	**FAM159A**	3.92	2.52E-05	2.45	0.00053772	1.87	0.01695014	2.48	0.00056453	2.58	0.00052949
**205239_at**	**AREG**	3.91	6.52E-10	2.61	8.75E-09	1.03	0.68193313	3.64	9.32E-10	4.73	2.70E-10
**212236_x_at**	**KRT17**	3.83	2.19E-08	2.52	3.38E-07	-1.02	0.88498677	3.63	3.02E-08	3.53	4.80E-08
**220370_s_at**	**USP36**	3.82	1.29E-08	3.25	3.08E-08	1.21	0.04597236	3.25	3.33E-08	2.95	8.79E-08
**206157_at**	**PTX3**	3.81	7.73E-10	2.46	1.43E-08	-1.11	0.10310023	3.61	1.04E-09	3.78	9.68E-10
**1555571_at**	**IMMP2L**	3.81	1.54E-07	3.89	1.19E-07	2.71	1.41E-05	4.10	1.00E-07	3.37	4.69E-07
**1555777_at**	**POSTN**	3.80	7.36E-08	3.60	8.95E-08	2.44	1.42E-05	3.79	7.42E-08	2.83	7.38E-07
**224978_s_at**	**USP36**	3.70	2.36E-09	3.53	3.53E-09	1.41	0.00039992	3.40	4.14E-09	3.52	4.06E-09
**214508_x_at**	**CREM**	3.69	3.04E-09	3.06	9.50E-09	-1.01	0.88661058	3.34	5.68E-09	3.59	4.49E-09
**1554805_at**	**CLDN19**	3.65	3.83E-07	2.98	1.25E-06	1.07	0.66920298	3.38	6.07E-07	2.57	6.53E-06
**223551_at**	**PKIB**	3.63	1.43E-09	3.98	9.54E-10	-1.11	0.10975525	3.01	4.96E-09	3.32	3.02E-09
**203984_s_at**	**CASP9**	3.59	1.18E-09	3.77	9.54E-10	1.07	0.27550502	3.24	2.13E-09	3.37	2.00E-09
**207630_s_at**	**CREM**	3.55	2.36E-08	2.87	8.76E-08	-1.03	0.78865521	3.19	4.69E-08	3.40	4.19E-08

		NPSR1		NPSR1-107Asn		NPSR1-197Phe		NPSR1-241Ser		NPSR1-344Arg	
**Affy probeset**	**Gene**	**fold change**	**p value**	**fold change**	**p value**	**fold change**	**p value**	**fold change**	**p value**	**fold change**	**p value**
**209636_at**	**NFKB2**	3.52	1.19E-08	3.27	1.67E-08	*1.86*	*2.04E-05*	3.33	1.64E-08	3.31	2.24E-08
**206374_at**	**DUSP8**	3.44	9.60E-08	2.89	2.73E-07	*1.42*	*0.00598562*	3.35	1.08E-07	2.82	5.36E-07
**225699_at**	**C7orf40**	3.43	1.75E-09	3.29	2.47E-09	2.12	1.16E-06	3.39	1.90E-09	2.63	1.52E-08
**207574_s_at**	**GADD45B**	3.41	7.70E-10	3.13	1.43E-09	2.09	7.00E-07	3.53	5.94E-10	3.00	2.00E-09
**210764_s_at**	**CYR61**	3.40	2.66E-07	2.21	7.59E-06	*1.07*	*0.62216285*	3.36	2.78E-07	3.47	3.28E-07
**202081_at**	**IER2**	3.37	8.82E-10	2.75	3.74E-09	*1.20*	*0.00662729*	3.11	1.50E-09	3.07	1.90E-09
**226345_at**	**NA**	3.34	2.04E-07	3.19	2.41E-07	2.38	2.09E-05	3.19	2.76E-07	3.14	4.30E-07
**65588_at**	**LOC388796**	3.34	2.30E-07	3.02	4.05E-07	2.12	6.99E-05	3.23	2.78E-07	2.51	2.99E-06
**1558290_a_at**	**PVT1**	3.34	1.41E-08	3.47	1.06E-08	2.63	1.16E-06	3.59	9.00E-09	2.30	3.28E-07
**209304_x_at**	**GADD45B**	3.29	8.25E-09	2.98	1.43E-08	*1.97*	*6.35E-06*	3.38	6.58E-09	2.85	2.84E-08
**205205_at**	**RELB**	3.29	1.41E-08	3.12	1.83E-08	*1.93*	*1.29E-05*	3.12	2.12E-08	2.53	1.26E-07
**201041_s_at**	**DUSP1**	3.28	8.68E-09	2.55	4.59E-08	*1.10*	*0.20407608*	3.16	1.08E-08	3.48	7.60E-09
**201289_at**	**CYR61**	3.25	2.24E-09	2.34	2.47E-08	*1.25*	*0.00324078*	3.42	1.77E-09	3.33	2.28E-09
**207371_at**	**NA**	3.21	1.24E-07	2.68	4.05E-07	*1.97*	*6.44E-05*	3.11	1.46E-07	2.40	1.71E-06
**217127_at**	**CTH**	3.20	4.70E-09	3.17	5.11E-09	*1.60*	*3.55E-05*	3.10	5.89E-09	2.72	1.94E-08
**218371_s_at**	**PSPC1**	3.20	2.01E-09	3.15	2.47E-09	2.29	6.13E-07	3.28	1.77E-09	2.46	1.91E-08
**241985_at**	**JMY**	3.20	3.66E-08	3.01	4.92E-08	*1.15*	*0.13339293*	2.86	7.73E-08	2.97	8.45E-08
**204420_at**	**FOSL1**	3.18	5.70E-08	2.18	1.13E-06	*1.10*	*0.3188206*	3.02	7.78E-08	3.12	8.98E-08
**221128_at**	**ADAM19**	3.17	1.71E-06	2.08	6.12E-05	*1.05*	*0.77811731*	3.46	9.14E-07	3.38	1.56E-06
**214446_at**	**ELL2**	3.15	7.06E-08	2.52	3.26E-07	*-1.31*	*0.01246209*	2.83	1.39E-07	3.53	4.63E-08
**222612_at**	**PSPC1**	3.14	6.96E-09	3.13	7.20E-09	2.09	2.24E-06	3.26	5.49E-09	2.33	8.86E-08
**207536_s_at**	**TNFRSF9**	3.13	6.22E-06	2.28	7.58E-05	*1.46*	*0.03511983*	3.51	2.61E-06	3.01	1.03E-05
**218368_s_at**	**TNFRSF12A**	3.13	2.76E-06	2.43	1.84E-05	*1.38*	*0.04494261*	3.45	1.33E-06	2.99	5.11E-06
**214636_at**	**CALCB**	3.12	1.88E-08	2.46	9.58E-08	*1.02*	*0.81272719*	2.57	7.69E-08	3.00	3.33E-08
**1565495_at**	**NA**	3.12	0.00026918	2.81	0.00045017	*1.64*	*0.06973869*	3.35	0.00016125	2.28	0.00297707
**220908_at**	**CCDC33**	3.10	4.91E-07	2.75	1.03E-06	*1.74*	*0.00063053*	2.77	1.10E-06	2.50	3.74E-06
**219228_at**	**ZNF331**	3.10	1.41E-08	2.83	2.47E-08	*1.23*	*0.01472005*	2.68	4.08E-08	2.87	3.29E-08
**207850_at**	**CXCL3**	3.06	2.17E-09	2.50	9.50E-09	*1.05*	*0.3917555*	3.11	1.96E-09	2.59	9.53E-09
**209967_s_at**	**CREM**	3.06	1.43E-09	2.60	4.83E-09	*-1.03*	*0.56773739*	2.72	3.39E-09	2.95	2.14E-09
**205157_s_at**	**KRT17**	3.06	2.19E-08	2.13	3.92E-07	*1.05*	*0.534791*	2.83	3.83E-08	2.79	5.74E-08
**230815_at**	**LOC389765**	3.04	1.38E-08	3.29	8.13E-09	2.39	1.18E-06	3.02	1.42E-08	2.32	1.52E-07
**208891_at**	**DUSP6**	3.02	4.73E-09	2.54	1.64E-08	*1.01*	*0.91418939*	3.13	3.92E-09	3.02	5.86E-09
**235200_at**	**NA**	3.01	4.16E-05	3.17	2.34E-05	2.10	0.00298601	3.06	3.68E-05	2.60	0.00015454
**225857_s_at**	**LOC388796**	2.99	7.31E-09	2.86	9.50E-09	2.10	1.80E-06	3.02	6.58E-09	2.32	6.97E-08
**228200_at**	**ZNF252**	2.94	3.05E-08	2.62	6.77E-08	*1.88*	*1.68E-05*	2.74	5.40E-08	2.43	1.98E-07

Affy probeset	Gene	NPSR1 fold change	NPSR1 p value	NPSR1-107Asn fold change	NPSR1-107Asn p value	NPSR1-197Phe fold change	NPSR1-197Phe p value	NPSR1-241Ser fold change	NPSR1-241Ser p value	NPSR1-344Arg fold change	NPSR1-344Arg p value
**202409_at**	**NA**	2.93	2.32E-07	2.84	2.57E-07	2.39	1.18E-05	3.17	1.29E-07	2.02	9.53E-06
**244884_at**	**NA**	2.93	3.07E-06	2.73	4.49E-06	2.04	0.00038794	2.89	3.25E-06	2.16	5.86E-05
**209795_at**	**CD69**	2.92	3.32E-08	2.06	6.15E-07	*1.19*	*0.04606895*	3.01	2.80E-08	2.96	4.19E-08
**204829_s_at**	**FOLR2**	2.92	1.17E-07	2.81	1.35E-07	*1.64*	*0.00030204*	2.77	1.69E-07	2.09	3.25E-06
**230821_at**	**ZNF148**	2.91	0.0009237	*1.90*	*0.01608298*	*1.10*	*0.78865521*	2.06	0.00984224	*1.92*	*0.01926072*
**209239_at**	**NFKB1**	2.89	8.35E-08	2.41	3.12E-07	*1.33*	*0.00779544*	2.78	1.07E-07	2.89	1.14E-07
**227301_at**	**NA**	2.88	1.29E-08	3.13	7.20E-09	2.41	8.94E-07	3.02	8.82E-09	2.20	1.58E-07
**201044_x_at**	**DUSP1**	2.86	1.25E-05	*1.78*	*0.00099966*	*-1.03*	*0.88229636*	2.65	2.27E-05	3.08	8.75E-06
**220225_at**	**IRX4**	2.85	3.73E-08	3.07	2.14E-08	*-1.03*	*0.78243673*	2.49	1.04E-07	2.51	1.40E-07
**209386_at**	**TM4SF1**	2.83	6.25E-09	*1.48*	*1.15E-05*	*-1.01*	*0.89259753*	2.38	2.41E-08	3.85	1.01E-09
**208127_s_at**	**SOCS5**	2.83	2.71E-07	2.52	6.11E-07	*1.61*	*0.00061022*	2.48	8.12E-07	2.09	6.11E-06
**1555411_a_at**	**CCNL1**	2.79	4.18E-08	2.53	8.64E-08	*1.46*	*0.0005494*	2.64	6.68E-08	2.36	2.41E-07
**209305_s_at**	**GADD45B**	2.79	1.34E-06	2.36	4.74E-06	*1.67*	*0.00131079*	2.91	9.14E-07	2.59	3.34E-06
**203499_at**	**EPHA2**	2.76	1.41E-08	2.18	8.95E-08	*-1.02*	*0.84919827*	2.74	1.55E-08	2.77	1.89E-08
**227613_at**	**ZNF331**	2.75	1.91E-08	2.55	3.27E-08	*1.09*	*0.2162438*	2.37	6.51E-08	2.37	8.86E-08
**220046_s_at**	**CCNL1**	2.74	2.86E-08	2.67	3.27E-08	*1.61*	*6.99E-05*	2.83	2.40E-08	2.43	1.03E-07
**205931_s_at**	**CREB5**	2.73	4.15E-07	2.32	1.42E-06	*1.40*	*0.00668472*	2.64	5.05E-07	2.41	1.66E-06
**205290_s_at**	**BMP2**	2.71	5.02E-07	*1.82*	*2.72E-05*	*1.17*	*0.14269225*	2.44	1.21E-06	3.22	2.00E-07
**231879_at**	**COL12A1**	2.71	1.74E-06	2.42	3.95E-06	*1.86*	*0.00038146*	2.62	2.22E-06	2.48	5.11E-06
**219153_s_at**	**THSD4**	2.70	1.11E-06	2.06	1.23E-05	*1.45*	*0.0072545*	2.50	2.09E-06	*1.92*	*4.39E-05*
**241058_at**	**NA**	2.66	6.34E-05	2.32	0.00017522	*1.56*	*0.03028556*	2.44	0.00012447	*1.70*	*0.00574055*
**203023_at**	**NOP16**	2.66	9.54E-07	2.33	2.60E-06	*1.80*	*0.00030204*	2.71	8.07E-07	2.39	3.35E-06
**223218_s_at**	**NFKBIZ**	2.62	2.45E-08	*1.91*	*4.72E-07*	*1.03*	*0.76269876*	2.59	2.86E-08	2.46	5.76E-08
**211458_s_at**	**NA**	2.62	4.65E-09	2.34	1.06E-08	*1.16*	*0.01577439*	2.60	4.96E-09	2.69	4.49E-09
**203349_s_at**	**ETV5**	2.62	8.25E-09	2.23	3.00E-08	*-1.01*	*0.9078578*	2.46	1.41E-08	2.54	1.39E-08
**1562629_a_at**	**KRT40**	2.61	1.16E-06	2.18	5.42E-06	*1.77*	*0.00037151*	2.42	2.20E-06	2.35	4.14E-06
**214016_s_at**	**SFPQ**	2.60	1.59E-07	2.77	8.95E-08	2.18	8.25E-06	2.60	1.54E-07	*1.95*	*4.10E-06*
**230511_at**	**CREM**	2.58	1.29E-08	2.14	6.08E-08	*1.00*	*0.99235469*	2.31	3.18E-08	2.45	2.60E-08
**214944_at**	**PHLPP2**	2.58	5.12E-05	2.21	0.00016775	*1.66*	*0.01289169*	2.40	9.06E-05	*1.87*	*0.00135933*
**227770_at**	**NA**	2.57	9.97E-07	2.52	9.90E-07	*1.96*	*9.22E-05*	2.43	1.56E-06	2.54	1.52E-06
**205825_at**	**PCSK1**	2.57	2.06E-07	2.61	1.57E-07	*1.07*	*0.47204027*	2.34	4.72E-07	2.59	2.71E-07
**206381_at**	**SCN2A**	2.56	2.82E-05	2.19	9.99E-05	*1.61*	*0.01172219*	2.35	5.89E-05	*1.91*	*0.00061121*
**225688_s_at**	**PHLDB2**	2.55	5.21E-06	2.47	5.71E-06	*1.03*	*0.8511207*	2.66	3.38E-06	2.81	3.12E-06
**204602_at**	**DKK1**	2.54	4.18E-08	*1.83*	*1.11E-06*	*-1.07*	*0.38927328*	2.35	8.23E-08	2.83	2.57E-08
**206036_s_at**	**REL**	2.54	2.52E-06	2.20	8.07E-06	*1.13*	*0.32260783*	2.69	1.45E-06	2.37	6.10E-06

		NPSR1		NPSR1-107Asn		NPSR1-197Phe		NPSR1-241Ser		NPSR1-344Arg	
**Affy probeset**	**Gene**	**fold change**	**p value**	**fold change**	**p value**	**fold change**	**p value**	**fold change**	**p value**	**fold change**	**p value**
**213855_s_at**	**LIPE**	2.54	9.28E-05	2.25	0.00022077	1.57	0.02865454	2.63	6.68E-05	1.74	0.00436126
**202332_at**	**CSNK1E**	2.53	6.89E-09	2.36	1.10E-08	2.38	2.88E-07	2.60	5.49E-09	2.56	7.80E-09
**216350_s_at**	**ZNF10**	2.53	3.77E-08	2.21	1.10E-07	-1.03	0.70732808	2.28	9.18E-08	2.34	1.02E-07
**230086_at**	**FNBP1**	2.53	2.31E-06	2.35	3.79E-06	1.27	0.05730547	2.06	1.83E-05	1.77	0.00015154
**219679_s_at**	**WAC**	2.52	1.36E-06	2.08	7.59E-06	2.03	7.53E-05	2.37	2.29E-06	2.54	1.73E-06
**219248_at**	**THUMPD2**	2.51	2.73E-08	2.60	2.00E-08	2.03	2.28E-06	2.52	2.80E-08	2.03	2.87E-07
**227337_at**	**ANKRD37**	2.51	7.15E-06	2.03	5.22E-05	1.15	0.31620555	2.27	1.86E-05	2.23	2.72E-05
**1561305_at**	**NA**	2.50	6.07E-07	2.10	2.87E-06	1.52	0.00124417	2.56	4.81E-07	1.87	1.73E-05
**221580_s_at**	**TAF1D**	2.49	5.20E-08	2.59	3.69E-08	2.00	4.78E-06	2.55	4.43E-08	2.09	3.87E-07
**208383_s_at**	**PCK1**	2.48	2.79E-06	1.69	0.00019937	-1.27	0.05730547	1.83	7.89E-05	2.99	8.52E-07
**230251_at**	**C6orf176**	2.47	2.45E-08	2.25	5.04E-08	1.02	0.78075969	2.24	5.93E-08	2.44	3.78E-08
**204260_at**	**CHGB**	2.46	3.73E-08	2.83	1.17E-08	1.20	0.01760803	2.30	6.63E-08	2.74	2.17E-08
**1565677_at**	**NA**	2.46	7.25E-05	2.37	8.33E-05	1.69	0.01028419	2.56	4.86E-05	2.00	0.00063608
**202531_at**	**IRF1**	2.45	3.52E-08	2.29	5.74E-08	2.00	2.60E-06	2.50	3.02E-08	2.25	1.01E-07
**234113_at**	**NA**	2.44	7.06E-08	2.36	8.76E-08	1.81	1.42E-05	2.08	3.27E-07	1.72	5.18E-06
**226618_at**	**FLJ25076**	2.44	1.36E-07	2.15	3.92E-07	1.18	0.05533872	2.25	2.78E-07	2.51	1.46E-07
**201631_s_at**	**IER3**	2.43	1.91E-08	2.08	7.77E-08	1.46	9.81E-05	2.72	7.78E-09	2.33	3.81E-08
**201693_s_at**	**EGR1**	2.43	2.62E-05	1.92	0.00023568	1.03	0.85642495	2.15	8.30E-05	2.11	0.00012737
**209101_at**	**CTGF**	2.42	1.01E-07	1.82	1.93E-06	1.02	0.78671753	2.58	6.03E-08	2.65	6.66E-08
**206085_s_at**	**CTH**	2.41	7.28E-07	2.25	1.15E-06	1.34	0.01095188	2.34	9.14E-07	2.22	2.20E-06
**201645_at**	**TNC**	2.41	4.80E-06	1.76	0.00014736	-1.11	0.38670317	2.32	6.63E-06	2.68	2.53E-06
**203348_s_at**	**ETV5**	2.40	1.60E-08	1.99	8.95E-08	1.05	0.46089758	2.32	2.27E-08	2.62	1.06E-08
**225262_at**	**FOSL2**	2.40	4.65E-08	2.17	1.04E-07	1.14	0.06413176	2.26	7.78E-08	2.24	1.23E-07
**229799_s_at**	**NCAM1**	2.39	9.16E-05	2.29	0.0001144	1.60	0.01697547	2.43	7.64E-05	1.90	0.00110081
**227208_at**	**CCDC84**	2.39	1.43E-07	2.28	1.91E-07	1.85	1.84E-05	2.32	1.81E-07	1.70	1.05E-05
**1557558_s_at**	**LOC1001291**	2.38	1.44E-08	2.59	7.39E-09	1.92	1.80E-06	2.47	1.08E-08	1.83	3.31E-07
**209553_at**	**VPS8**	2.38	2.68E-06	2.07	9.18E-06	1.47	0.00519814	2.15	7.30E-06	1.77	9.86E-05
**210775_x_at**	**CASP9**	2.37	1.15E-07	2.42	8.95E-08	1.07	0.3917555	2.11	3.49E-07	2.31	2.12E-07
**204470_at**	**CXCL1**	2.37	1.13E-07	1.95	7.47E-07	1.18	0.03895091	2.40	9.81E-08	1.98	1.02E-06
**228812_at**	**NA**	2.37	1.99E-06	2.24	2.87E-06	1.13	0.27120163	2.27	2.88E-06	2.22	5.11E-06
**211564_s_at**	**PDLIM4**	2.37	4.92E-07	2.13	1.19E-06	1.10	0.31628571	2.17	1.12E-06	1.89	7.66E-06
**201925_s_at**	**CD55**	2.37	8.93E-08	1.96	5.43E-07	1.01	0.87813031	2.16	2.04E-07	2.61	5.45E-08
**224489_at**	**KIAA1267**	2.36	7.06E-08	2.16	1.40E-07	1.29	0.00407066	2.27	9.96E-08	1.66	6.20E-06
**213315_x_at**	**CXorf40A**	2.35	2.05E-08	2.18	3.73E-08	1.09	0.16363711	2.21	3.58E-08	2.16	6.19E-08
**224979_s_at**	**USP36**	2.35	4.73E-07	2.16	8.87E-07	1.23	0.03129568	2.01	2.26E-06	2.06	2.51E-06

		NPSR1		NPSR1-107Asn		NPSR1-197Phe		NPSR1-241Ser		NPSR1-344Arg	
Affy probeset	Gene	fold change	p value	fold change	p value	fold change	p value	fold change	p value	fold change	p value
203574_at	NFIL3	2.34	7.06E-08	2.46	4.27E-08	1.20	0.01862257	2.23	1.07E-07	2.13	2.44E-07
1566475_at	NA	2.34	1.57E-07	2.11	3.92E-07	1.28	0.00748191	1.99	8.60E-07	1.72	7.86E-06
240630_at	NA	2.33	3.67E-05	1.76	0.00067102	1.29	0.0984019	1.87	0.000368	1.45	0.01273912
203862_s_at	ACTN2	2.33	3.46E-06	2.48	1.58E-06	1.71	0.00057056	2.27	4.22E-06	2.12	1.17E-05
207535_s_at	NFKB2	2.31	1.85E-06	2.00	7.41E-06	1.44	0.00424505	2.32	1.76E-06	2.16	5.11E-06
226034_at	NA	2.31	1.49E-08	1.95	8.15E-08	1.10	0.09140747	2.24	2.21E-08	2.48	1.06E-08
226612_at	FLJ25076	2.31	2.58E-08	2.14	4.68E-08	1.12	0.07249048	2.34	2.40E-08	2.46	2.02E-08
241459_at	LIMCH1	2.30	1.27E-06	2.23	1.44E-06	1.69	0.0002907	2.27	1.39E-06	1.95	1.01E-05
213820_s_at	STARD5	2.29	0.0032952	1.98	0.00806103	1.36	0.27688129	2.31	0.00288697	1.48	0.10624274
220987_s_at	NA	2.28	2.97E-08	2.05	8.15E-08	1.27	0.00207099	2.37	2.27E-08	2.10	9.16E-08
242329_at	NA	2.28	2.07E-07	2.17	2.96E-07	1.48	0.0003922	2.49	9.18E-08	1.87	2.87E-06
218332_at	BEX1	2.28	3.46E-07	2.35	2.25E-07	1.53	0.00037151	2.31	2.89E-07	1.72	1.32E-05
229848_at	ZNF10	2.28	5.30E-06	2.13	9.05E-06	1.04	0.78671753	2.02	1.99E-05	2.27	7.15E-06
220578_at	ADAMTSL4	2.28	1.71E-06	2.07	3.94E-06	1.55	0.00103756	2.16	2.80E-06	1.92	1.49E-05
204491_at	PDE4D	2.28	1.25E-05	2.23	1.23E-05	1.16	0.2557697	2.01	4.76E-05	2.17	2.53E-05
208869_s_at	GABARAPL1	2.27	2.09E-06	1.98	8.11E-06	1.11	0.32971599	2.29	1.85E-06	2.58	9.21E-07
201502_s_at	NFKBIA	2.26	3.95E-08	2.02	1.10E-07	1.47	9.22E-05	2.38	2.73E-08	1.98	2.34E-07
205680_at	MMP10	2.26	2.36E-08	1.31	0.00013795	-1.02	0.79294217	1.92	1.22E-07	3.14	2.14E-09
212961_x_at	CXorf40B	2.26	2.76E-08	2.11	4.87E-08	1.09	0.16762987	2.14	4.81E-08	2.12	7.06E-08
211996_s_at	NA	2.25	0.00184403	2.55	0.00055658	2.02	0.01289169	2.23	0.0019016	1.96	0.00677539
222728_s_at	TAF1D	2.25	1.60E-07	2.25	1.40E-07	1.92	9.15E-06	2.42	8.23E-08	1.96	1.07E-06
1561676_at	NA	2.25	7.15E-06	1.76	0.0001139	1.33	0.02753592	1.76	0.00012538	1.53	0.00131599
221667_s_at	HSPB8	2.25	1.41E-07	1.90	7.66E-07	1.21	0.01845731	2.25	1.35E-07	2.10	3.97E-07
221704_s_at	VPS37B	2.23	7.15E-06	2.09	1.16E-05	1.13	0.32260783	2.19	8.48E-06	2.01	2.81E-05
202147_s_at	IFRD1	2.22	9.81E-07	1.97	3.23E-06	1.09	0.35441749	2.03	2.41E-06	1.99	4.61E-06
230512_x_at	TMEM165	2.22	4.91E-07	2.01	1.21E-06	1.52	0.00042865	2.29	3.45E-07	1.79	8.11E-06
204346_s_at	RASSF1	2.22	7.99E-07	2.22	6.58E-07	1.76	7.60E-05	2.31	5.05E-07	1.87	7.19E-06
202861_at	PER1	2.22	9.47E-07	2.24	7.23E-07	1.41	0.0028169	2.03	2.29E-06	1.91	6.72E-06
210910_s_at	POMZP3	2.22	1.45E-07	2.28	9.84E-08	1.81	1.31E-05	2.24	1.28E-07	1.64	8.54E-06
205483_s_at	ISG15	2.21	1.83E-07	2.10	2.73E-07	1.11	0.19030631	2.18	2.09E-07	1.95	1.04E-06
206237_s_at	NRG1	2.21	1.32E-07	1.86	8.02E-07	1.08	0.29306749	1.96	4.80E-07	2.08	3.42E-07
214448_x_at	NFKBIB	2.20	1.07E-07	2.01	2.37E-07	1.42	0.000362	2.15	1.28E-07	1.78	1.74E-06
202149_at	NEDD9	2.19	1.88E-05	1.89	8.31E-05	-1.06	0.68193313	2.05	3.79E-05	2.19	2.33E-05
214112_s_at	NA	2.19	1.46E-08	2.12	1.98E-08	1.13	0.03238561	2.12	2.21E-08	1.99	5.45E-08
235242_at	NA	2.19	3.71E-05	1.95	0.00010803	1.04	0.78865521	2.17	3.85E-05	2.22	3.88E-05

		NPSR1		NPSR1-107Asn		NPSR1-197Phe		NPSR1-241Ser		NPSR1-344Arg	
Affy probeset	Gene	fold change	p value	fold change	p value	fold change	p value	fold change	p value	fold change	p value
1570031_at	NA	2.18	3.86E-05	2.01	7.70E-05	1.55	0.00825771	2.17	3.89E-05	1.78	0.00047393
223939_at	SUCNR1	2.17	1.60E-06	2.09	1.96E-06	1.48	0.00138075	2.27	9.47E-07	1.71	4.21E-05
229621_x_at	NA	2.16	0.00019018	1.80	0.00108038	1.46	0.03776322	1.95	0.00055294	1.49	0.01505075
227173_s_at	BACH2	2.16	7.82E-06	2.21	4.89E-06	1.22	0.087927	2.19	6.47E-06	2.30	5.18E-06
203556_at	ZHX2	2.16	2.03E-05	2.28	9.12E-06	1.08	0.6054141	1.95	6.07E-05	2.07	3.86E-05
214062_x_at	NFKBIB	2.16	3.71E-08	2.14	3.80E-08	1.56	2.09E-05	2.15	3.99E-08	1.84	3.04E-07
201110_s_at	THBS1	2.15	4.51E-07	1.39	0.00032708	-1.08	0.35631574	2.32	2.00E-07	2.38	2.36E-07
204435_at	NUPL1	2.14	2.46E-05	2.10	2.38E-05	1.33	0.03896289	1.97	5.95E-05	2.02	5.45E-05
209621_s_at	PDLIM3	2.14	2.09E-07	1.93	5.57E-07	-1.01	0.88661058	2.07	2.90E-07	2.07	4.16E-07
219425_at	SULT4A1	2.13	3.46E-07	2.33	1.22E-07	1.55	0.00015895	2.17	2.78E-07	1.79	3.97E-06
233964_at	NA	2.13	7.33E-05	1.91	0.00020468	1.39	0.0365953	2.07	9.71E-05	1.57	0.00341127
201531_at	ZFP36	2.13	1.10E-07	1.68	1.85E-06	1.04	0.5701009	2.12	1.11E-07	1.96	3.80E-07
215009_s_at	SEC31A	2.13	5.91E-07	2.13	4.79E-07	1.47	0.00058362	2.19	4.15E-07	1.83	4.80E-06
219270_at	CHAC1	2.12	3.92E-06	2.06	4.52E-06	1.47	0.00257032	2.14	3.33E-06	1.65	0.00012439
203000_at	STMN2	2.11	8.45E-05	1.94	0.00017565	1.36	0.05213555	2.17	6.18E-05	1.84	0.0004816
205409_at	FOSL2	2.10	1.40E-07	1.85	5.57E-07	1.13	0.07775728	1.91	4.15E-07	1.83	1.05E-06
202302_s_at	RSRC2	2.10	1.60E-07	2.06	1.81E-07	1.49	0.00015032	2.06	1.98E-07	1.82	1.31E-06
204015_s_at	DUSP4	2.10	9.60E-08	1.93	2.18E-07	1.26	0.00353623	2.22	5.50E-08	2.27	6.01E-08
224490_s_at	KIAA1267	2.10	1.73E-06	2.08	1.57E-06	1.21	0.04699172	2.18	1.06E-06	1.68	3.88E-05
208295_x_at	CSHL1	2.10	0.00039664	1.94	0.00071267	2.27	0.00070402	2.66	4.54E-05	1.94	0.00103133
223394_at	SERTAD1	2.10	1.35E-06	1.75	1.15E-05	1.19	0.05755929	2.11	1.17E-06	2.01	2.99E-06
226337_at	GORAB	2.09	3.92E-06	2.29	1.22E-06	1.64	0.00043323	2.11	3.37E-06	1.81	2.81E-05
213649_at	SFRS7	2.09	1.60E-06	2.18	8.18E-07	1.89	3.42E-05	2.17	9.91E-07	1.85	9.20E-06
204222_s_at	GLIPR1	2.09	2.30E-06	1.36	0.00172426	-1.07	0.4797581	2.32	7.56E-07	2.51	5.11E-07
220820_at	NA	2.09	6.16E-05	2.00	8.27E-05	1.23	0.1539052	2.04	7.72E-05	1.54	0.00331574
202146_at	IFRD1	2.08	2.44E-06	1.93	4.95E-06	1.10	0.33657184	1.96	4.73E-06	1.85	1.31E-05
204014_at	DUSP4	2.07	9.47E-07	1.78	5.07E-06	1.09	0.29953337	2.06	9.25E-07	2.51	1.98E-07
236850_at	CAPRIN1	2.07	8.76E-06	1.78	4.87E-05	1.18	0.13160797	1.78	5.66E-05	1.36	0.00577259
219627_at	ZNF767	2.07	8.96E-08	2.31	2.82E-08	2.20	8.94E-07	2.15	5.93E-08	1.58	4.80E-06
1568781_at	NA	2.06	3.92E-06	2.26	1.21E-06	1.66	0.00034758	2.19	1.92E-06	1.74	4.30E-05
235318_at	FBN1	2.06	1.06E-07	1.77	6.11E-07	1.35	0.00057056	1.95	1.96E-07	1.57	6.20E-06
229428_at	LOC1001324[illegible]	2.06	2.44E-07	2.21	1.02E-07	1.58	6.46E-05	2.03	2.77E-07	1.66	6.10E-06
207285_x_at	CSHL1	2.05	0.00016337	1.86	0.00041319	2.28	0.00027507	2.54	1.99E-05	2.06	0.00019416
218617_at	TRIT1	2.05	6.68E-07	2.31	1.66E-07	1.92	1.29E-05	2.11	4.69E-07	1.63	1.96E-05
241990_at	RHOV	2.05	0.00028576	1.80	0.00099501	1.39	0.05879579	2.04	0.00028986	1.74	0.00208734

		NPSR1		NPSR1-107Asn		NPSR1-197Phe		NPSR1-241Ser		NPSR1-344Arg	
**Affy probeset**	**Gene**	**fold change**	**p value**	**fold change**	**p value**	**fold change**	**p value**	**fold change**	**p value**	**fold change**	**p value**
202301_s_at	RSRC2	2.04	5.95E-07	2.00	6.20E-07	1.47	0.00039992	2.00	7.39E-07	1.84	2.99E-06
228519_x_at	CIRBP	2.04	4.29E-06	2.29	1.03E-06	2.00	3.42E-05	2.34	9.25E-07	1.47	0.00067274
232960_at	NUP62CL	2.04	8.31E-06	1.86	2.14E-05	1.35	0.01437474	2.15	4.29E-06	1.76	6.65E-05
232352_at	ISL2	2.04	5.11E-07	2.02	4.72E-07	1.29	0.00505156	2.04	4.86E-07	1.79	3.53E-06
205251_at	PER2	2.03	7.06E-08	2.10	4.59E-08	1.18	0.0135681	2.09	5.50E-08	1.70	9.77E-07
209387_s_at	TM4SF1	2.03	9.08E-07	1.29	0.00192422	1.09	0.27887451	1.81	3.76E-06	2.54	1.39E-07
228736_at	HELQ	2.03	1.15E-05	2.16	4.74E-06	1.41	0.00825771	2.08	8.14E-06	1.79	6.64E-05
1559713_at	NA	2.03	0.00035946	1.70	0.00203132	1.48	0.03376719	1.94	0.00054791	1.58	0.00697406
1557026_at	NA	2.02	7.99E-05	1.74	0.00040404	1.40	0.02769507	1.82	0.00025802	1.72	0.00067274
205807_s_at	TUFT1	2.02	7.87E-08	1.72	5.30E-07	1.17	0.01880008	2.10	5.15E-08	1.87	2.71E-07
208370_s_at	RCAN1	2.01	4.66E-08	1.80	1.61E-07	-1.07	0.24207908	2.05	4.03E-08	2.01	6.73E-08
204148_s_at	NA	2.01	1.52E-07	2.11	8.64E-08	1.71	1.18E-05	2.11	9.18E-08	1.57	7.15E-06
208868_s_at	GABARAPL1	2.01	0.05057136	1.80	0.08083751	1.03	0.94209248	1.98	0.05292972	4.32	0.00109303
204157_s_at	QSK	2.00	1.68E-06	2.03	1.21E-06	1.26	0.01679394	1.99	1.74E-06	1.84	6.46E-06
221011_s_at	LBH	2.00	9.08E-07	1.82	2.50E-06	-1.15	0.0886091	2.03	7.41E-07	2.10	7.38E-07
232784_at	NA	1.99	0.00068397	1.85	0.00120779	1.26	0.21169107	2.12	0.00034839	1.55	0.01247731
230380_at	THAP2	1.99	4.40E-07	1.41	0.00010213	1.00	0.99614853	1.66	5.23E-06	2.07	3.83E-07
225932_s_at	HNRNPA2B1	1.99	4.16E-08	2.08	2.47E-08	1.87	1.18E-06	1.99	4.21E-08	1.62	9.77E-07
223430_at	SIK2	1.99	8.52E-06	2.02	5.65E-06	1.04	0.76364047	1.80	3.16E-05	1.89	1.99E-05
203725_at	GADD45A	1.99	8.85E-08	1.94	9.84E-08	1.32	0.00055031	2.04	6.56E-08	1.68	1.13E-06
212445_s_at	NEDD4L	1.97	9.01E-07	1.72	4.49E-06	1.23	0.01695014	1.86	1.72E-06	2.05	7.95E-07
228953_at	WHAMM	1.97	5.92E-07	2.17	1.73E-07	1.69	3.37E-05	1.99	5.05E-07	1.75	3.97E-06
214965_at	SPATA2L	1.96	2.26E-07	2.11	8.95E-08	1.41	0.00026856	2.05	1.36E-07	1.70	2.20E-06
213793_s_at	HOMER1	1.96	5.73E-07	1.75	2.15E-06	1.03	0.69542043	1.97	5.10E-07	2.19	2.36E-07
203140_at	BCL6	1.91	2.91E-06	1.84	3.91E-06	1.05	0.5701009	2.00	1.56E-06	2.05	1.66E-06
227093_at	USP36	1.90	8.19E-05	2.10	2.23E-05	1.28	0.06482699	1.87	9.90E-05	1.78	0.00023045
209824_s_at	ARNTL	1.90	1.16E-06	2.01	4.93E-07	1.55	0.00013575	1.95	8.12E-07	1.74	5.27E-06
209212_s_at	KLF5	1.90	9.61E-05	1.51	0.00184517	1.05	0.77811731	1.91	8.52E-05	2.03	5.47E-05
218750_at	TAF1D	1.89	2.69E-07	2.03	1.06E-07	1.65	1.61E-05	2.16	6.46E-08	1.63	3.29E-06
208293_x_at	CSHL1	1.89	0.00014032	1.73	0.00036746	1.88	0.00058362	2.44	8.83E-06	1.87	0.00019416
201324_at	EMP1	1.88	5.65E-06	1.54	0.00010839	-1.16	0.10962755	1.74	1.60E-05	2.14	1.64E-06
237755_s_at	WDR16	1.88	0.00540126	1.80	0.00644761	1.30	0.25705885	2.05	0.00233465	1.48	0.05640746
224833_at	ETS1	1.87	4.67E-05	1.58	0.00043367	1.02	0.87813031	2.01	1.96E-05	2.03	2.16E-05
225999_at	RIMKLB	1.87	1.29E-05	1.50	0.00031669	-1.08	0.46306256	1.54	0.00023338	2.19	2.68E-06
238592_at	PDLIM3	1.78	0.00015817	1.83	9.43E-05	1.03	0.83737917	1.85	9.44E-05	2.07	3.19E-05

		NPSR1		NPSR1-107Asn		NPSR1-197Phe		NPSR1-241Ser		NPSR1-344Arg	
**Affy probeset**	**Gene**	**fold change**	**p value**	**fold change**	**p value**	**fold change**	**p value**	**fold change**	**p value**	**fold change**	**p value**
**226651_at**	**HOMER1**	*1.76*	*2.91E-05*	*1.63*	*8.20E-05*	*-1.05*	*0.67602293*	*1.74*	*3.61E-05*	2.02	6.11E-06
**213953_at**	**KRT20**	*1.76*	*5.66E-05*	*1.65*	*0.00012784*	2.25	2.04E-05	*1.66*	*0.00013228*	*1.05*	*0.55156572*
**204970_s_at**	**MAFG**	*1.76*	*2.45E-06*	*1.65*	*5.41E-06*	*1.23*	*0.01418803*	2.02	3.89E-07	*1.70*	*5.18E-06*
**210587_at**	**INHBE**	*1.71*	*2.13E-06*	*2.00*	*2.25E-07*	2.04	2.28E-06	2.03	2.09E-07	*1.42*	*7.95E-05*
**203108_at**	**GPRC5A**	*1.67*	*6.94E-06*	*1.33*	*0.00053292*	*-1.12*	*0.1433592*	*1.56*	*2.37E-05*	2.04	6.61E-07
**223368_s_at**	**METTL11A**	*1.50*	*0.0074641*	2.11	9.76E-05	*1.39*	*0.04701855*	*1.62*	*0.00244202*	*1.31*	*0.05518931*
**204121_at**	**GADD45G**	*1.23*	*0.01711162*	*1.39*	*0.00089967*	2.69	1.95E-06	*1.37*	*0.00137669*	*-1.05*	*0.53878937*
**221530_s_at**	**BHLHE41**	*1.00*	*0.97948118*	*1.23*	*0.05663257*	2.07	0.00013406	*1.08*	*0.47302313*	*-1.08*	*0.46689824*
**233827_s_at**	**SUPT16H**	*-1.66*	*0.00491218*	-2.02	0.00043609	*-1.68*	*0.01178436*	*-1.89*	*0.00106549*	*-1.29*	*0.10333196*
**205321_at**	**EIF2S3**	*-1.73*	*0.00593592*	*-1.94*	*0.00144781*	*-1.60*	*0.03111288*	-2.04	0.0010686	*-1.39*	*0.06623461*
**221618_s_at**	**TAF9B**	*-1.82*	*0.00141591*	-2.06	0.00031806	*-1.66*	*0.01172219*	*-1.92*	*0.00075308*	*-1.50*	*0.01565391*
**203626_s_at**	**SKP2**	*-1.85*	*0.00743683*	-2.16	0.00154706	*-1.86*	*0.01760803*	-2.19	0.00156989	*-1.39*	*0.11003033*
**231862_at**	**CBX5**	*-1.87*	*0.00257365*	*-1.93*	*0.00145331*	*-1.46*	*0.06635708*	-2.14	0.00064663	*-1.47*	*0.03747869*
**241114_s_at**	**NA**	*-1.98*	*1.97E-05*	-2.14	6.52E-06	*-1.98*	*0.00010361*	-2.03	1.40E-05	-2.06	1.48E-05
**240929_at**	**NA**	-2.12	4.42E-07	-2.05	5.54E-07	-2.23	2.68E-06	-2.03	7.06E-07	-2.14	5.51E-07
**222505_at**	**LMBR1**	-2.15	0.00420929	*-1.99*	*0.00644791*	*-1.89*	*0.0276279*	-2.23	0.00291513	*-1.85*	*0.01565391*
**201008_s_at**	**TXNIP**	-2.19	0.00090108	-2.26	0.00054204	*-1.26*	*0.2890696*	-2.29	0.00057454	*-1.84*	*0.00520796*
**223535_at**	**NUDT12**	-2.35	4.33E-05	*-1.78*	*0.00069698*	*-1.32*	*0.0842722*	-2.19	8.52E-05	*-1.88*	*0.00053665*
**202291_s_at**	**MGP**	-2.40	1.34E-06	-2.38	1.21E-06	-2.40	1.25E-05	-2.32	1.77E-06	-2.12	6.23E-06
**1554524_a_at**	**OLFM3**	-2.42	1.57E-06	-2.46	1.10E-06	-2.63	7.45E-06	-2.70	5.58E-07	-2.53	1.40E-06
**226577_at**	**PSEN1**	-2.93	5.36E-07	-2.96	4.27E-07	-3.10	3.70E-06	-3.19	2.78E-07	-2.91	7.95E-07
